# Ultrahigh-Mass Resolution Mass Spectrometry Imaging with an Orbitrap Externally Coupled to a High-Performance Data Acquisition System

**DOI:** 10.1021/acs.analchem.3c04146

**Published:** 2023-12-21

**Authors:** Andrej Grgic, Konstantin O. Nagornov, Anton N. Kozhinov, Jesse A. Michael, Ian G.M. Anthony, Yury O. Tsybin, Ron M.A. Heeren, Shane R. Ellis

**Affiliations:** † The Maastricht MultiModal Molecular Imaging (M4I) Institute, Division of Imaging Mass Spectrometry (IMS), Maastricht University, 6229-ER Maastricht, Netherlands; ‡ Spectroswiss, 1015 Lausanne, Switzerland; § Molecular Horizons and School of Chemistry and Molecular Bioscience, University of Wollongong, Wollongong, New South Wales 2522, Australia

## Abstract

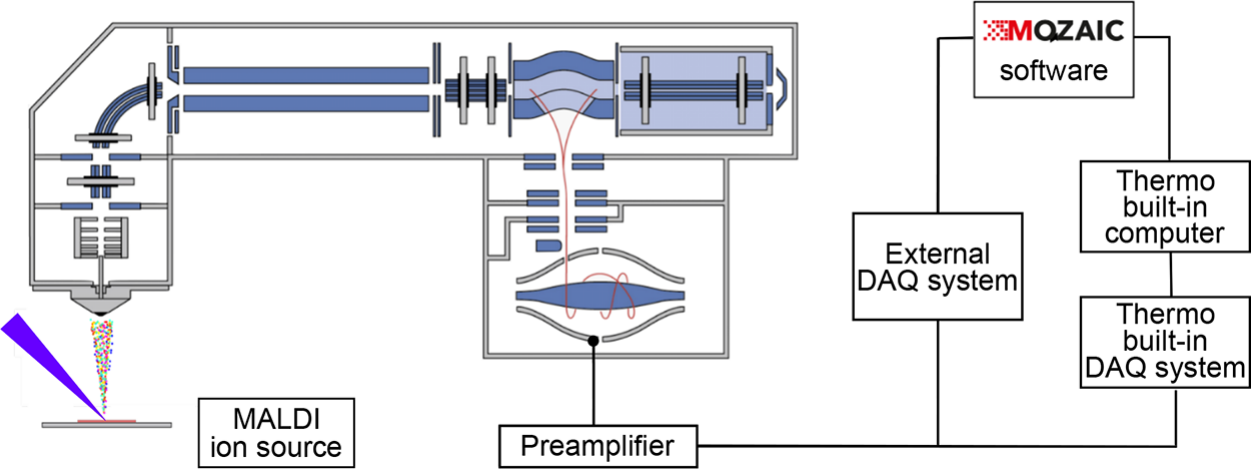

Matrix-assisted laser desorption ionization (MALDI) mass spectrometry imaging (MSI) is a powerful analytical tool that enables molecular sample analysis while simultaneously providing the spatial context of hundreds or even thousands of analytes. However, because of the lack of a separation step prior to ionization and the immense diversity of biomolecules, such as lipids, including numerous isobaric species, the coupling of ultrahigh mass resolution (UHR) with MSI presents one way in which this complexity can be resolved at the spectrum level. Until now, UHR MSI platforms have been restricted to Fourier transform ion cyclotron resonance (FT-ICR) mass spectrometers. Here, we demonstrate the capabilities of an Orbitrap-based UHR MSI platform to reach over 1,000,000 mass resolution in a lipid mass range (600–950 Da). Externally coupling the Orbitrap Q Exactive HF with the high-performance data acquisition system FTMS Booster X2 provided access to the unreduced data in the form of full-profile absorption-mode FT mass spectra. In addition, it allowed us to increase the time-domain transient length from 0.5 to 10 s, providing improvement in the mass resolution, signal-to-noise ratio, and mass accuracy. The resulting UHR performance generates high-quality MALDI MSI images and simplifies the identification of lipids. Collectively, these improvements resulted in a 1.5-fold increase in annotations, demonstrating the advantages of this UHR imaging platform for spatial lipidomics using MALDI-MSI.

## Introduction

Matrix-assisted laser desorption/ionization-mass spectrometry imaging (MALDI-MSI) enables the analysis of a sample’s molecular composition while simultaneously acquiring information on the spatial distribution of the molecules within it.^1^ The increasing popularity of MALDI-MSI stems from its versatility to image many different molecular classes, such as lipids, metabolites, pharmaceuticals, peptides, N-glycans, and intact proteins within tissue sections.^2^ MALDI-MSI can be applied to different sample types such as single cells, plant samples, and fresh frozen or formalin-fixed paraffin-embedded animal or human tissues.^2^

The chemical complexity of biological tissue samples can present a challenge for MSI as many signals may remain unresolved. For example, isobaric lipid ions, such as [13C2][PC 34:0 + Na]^+^ and [PC 34:1 + Na]^+^, may lead to inaccurate identification, quantification, and spatial mapping.^3,4^ Ultrahigh mass resolution (UHR) platforms, defined here as a mass resolution of over 500,000 at *m*/*z* 200 or exceeding 250,000 at *m*/*z* 800, may be able to resolve these isobaric signals. Orbitrap and ion cyclotron resonance (ICR) Fourier-transform (FT) mass spectrometers have demonstrated UHR performance.^4−7^ Recent advances in multireflectron time-of-flight instrumentation have also enhanced their mass resolving power and brought them to the UHR level (>200,000 in the lipid mass range).^8,9^ In addition to mass resolution, the ion mobility has also proven to be a powerful approach for resolving isobaric and some isomeric analytes, while recent developments in alternative ion activation techniques also enable the resolution of isomeric lipids that cannot be resolved with mass resolution alone.^10,11^

Recently, nanospray desorption electrospray ionization (nano-DESI), desorption electrospray ionization (DESI), laser ablation electrospray ionization (LAESI), and MALDI ion sources have been coupled with FT-ICR mass spectrometers and were shown to achieve mass resolution of 1,000,000 or more in the lipid mass range (600–950 Da).^4,12−16^ However, FT-ICR MS instruments, especially those with high magnetic field strengths, such as 18–21 T, are uncommon.

It has been demonstrated in proof-of-concept experiments that the first generation of Orbitraps, namely, LTQ Orbitrap XL instruments, may provide a mass resolution of up to 480,000 at *m*/*z* 800.^17^ To achieve this UHR performance, the LTQ Orbitrap XL was interfaced with the FTMS Booster X2, which is an external high-performance data acquisition and processing (DAQ/P) system.^17−19^ This enabled the acquisition of longer transients, access to the unreduced data (time-domain transients), and use of absorption-mode FT (aFT).^20−22^

In this work, we have coupled a MALDI MSI-enabled Orbitrap Q Exactive HF mass spectrometer to the FTMS Booster X2 for the acquisition and processing of signal transients independent of the Orbitrap data processing electronics.^23^ We demonstrate that, owing to the external high-performance DAQ/P system upgrade, the MALDI QE HF setup can perform UHR MSI measurements while simultaneously increasing the mass accuracy and sensitivity. The use of absorption-mode data representation combined with the ability to acquire long transients allowed for unrivaled mass spectral quality for the Orbitrap-based MSI platforms. We demonstrate that the developed UHR imaging platform achieves a mass resolution of over 1,000,000 in the lipid mass range (600–950 Da). This improvement resulted in an ∼1.6-fold increase in the number of observed peaks compared to the standard MALDI-MSI enabled Orbitrap QE HF. Furthermore, we show that increased mass accuracy combined with resolving the isotopic fine structure (IFS) leads to improved lipid identifications. Finally, these improvements combined resulted in an ∼1.5-fold increase in a number of annotations compared to that of the standard MALDI-enabled Orbitrap QE HF.

## Materials and Methods

### Chemicals

Water (HPLC MS grade), ethanol, methanol, and chloroform were obtained from Biosolve BV (Valkenswaard, Netherlands). Ammonium sulfate and 2,5-dihydroxyacetophenone (2,5-DHA) were acquired from Sigma-Aldrich (St. Louis, MI, USA).

### Samples

Fresh frozen mouse brain samples were obtained from the Johns Hopkins University School of Medicine. All animal experiments were performed with appropriate ethical approval (A3272–01 at Johns Hopkins University) and in compliance with the respective institutional guidelines.

### Sample Preparation

Mouse brain was sectioned to 12 μm of thickness with cryo-microtome (Leica, Nussloch, Germany) at −20 °C and thaw-mounted on indium tin oxide (ITO)-coated glass slides (Delta Technologies Ltd., Loveland, CO, USA). The slides were then stored at −80 °C until matrix application and MSI analysis.

Samples measured in the positive mode were prepared by spraying the norharmane matrix, while the sample measured in the negative mode was prepared by spraying a solution of 30 mg of 2,5-DHA and 40 mg of ammonium sulfate in 6 mL of 70% ethanol. An HTX M3+ sprayer (HTX Technologies, Carrboro, NC, USA) was used for all samples. The spraying parameters used can be found in Table S1.

### MALDI MSI

For each analysis, a prepared slide was placed into the MALDI ion source (Spectroglyph, Kennewick, WA, USA). The area to be measured was selected in MALDI injector software (Spectroglyph, v 1.3.1.964), and the pixel size was set between 50 × 50 μm and 70 × 70 μm.^24^ The *x* step of each pixel was set to half of the *y* step to compensate for the stage movement that occurred during each dummy scan. Additional information regarding the run time and strategies for its reduction can be accessed in the Supporting Information. This holds significance because the duration of MALDI-MSI experiments grows linearly with the length of the transient, resulting in a 14-fold increase in the measurement time for acquisitions with 7 s long transients.

### Coupling the MALDI-Enabled QE HF Orbitrap with the External Data Acquisition System

The QE HF (Thermo Fisher Scientific, Bremen, Germany) was externally interfaced with the high-performance DAQ/P system (FTMS Booster X2, Spectroswiss, Lausanne, Switzerland), as shown in Figure S1. The system was connected via the BNC-type T-junctions to the Orbitrap’s preamplifier’s two signal outputs.^23^ On the DAQ/P system, after on-board signal amplification and digitization, the digital transient signals were streamed through the on-board field programmable gate array (FPGA) chip for real-time digital signal processing and further forwarded to the embedded computer for final processing on the CPUs and file recording. The original built-in DAQ electronics of the Orbitrap was not modified and operated as usual, thus allowing for a direct comparison between two simultaneously acquired data sets. Thermo Xcalibur Instrument Setup software (version 4.2.47) was used to set up the method, while Instrument Control software (version 2.11 Build 3005) was used to control the mass spectrometer. To acquire transients with an extended duration, each analytically useful scan was followed by a dummy scan, as described elsewhere.^25^ The high-performance architecture of the FTMS Booster X2 allows us to decipher both the start trigger (ion injection into the orbitrap) and the stop trigger (ion ejection from the orbitrap) events, whereas the built-in DAQ system can only process the start trigger. As a result, data acquisition by the FTMS Booster X2 benefits from this dummy-scanning method because ions injected into the orbitrap (start trigger) keep oscillating while the next ion packet is being accumulated, allowing for a longer transient accumulation from a single ion packet (until the stop trigger).^19^ The stage moves once for the MS1 scan and once for the MSX sequence scan. The pixel corresponding to the dummy scan is filled through extrapolation from the nearest pixel, resulting in the generation of a square pixel.

For analytically useful scans, the all-ion fragmentation (AIF) scan mode was used with a mass range from *m*/*z* 650–900 in a positive ion mode and a mass range from *m*/*z* 500–3000 in a negative ion mode. The mass resolution for the RAW data was set to 120,000 in a positive mode and 240,000 in a negative mode (both at *m*/*z* 200). The normalized collision energy was set to a minimal value (N(CE) = 10) to minimize ion fragmentation. The automatic gain control (AGC) capability was turned off, and the number of charges collected was controlled by setting the maximum injection time to 550 ms. During dummy scans, very few MALDI-generated ions were accumulated as narrow mass ranges where no lipids or matrix ions were expected were selected. This forced the accumulation of ions for a set amount of time of up to 10 s. To enable the acquisition of extended transients, the vendor updated the Spectroglyph MALDI injector software (v 1.3.1.964) by delaying the safety trigger mechanism, which would previously terminate the run if it waited for the next trigger for longer than 3–10 s. The targeted–selected-ion monitoring (t-SIM) mode was used for the dummy scans. Settings for the t-SIM dummy scans were a mass resolution of 120,000 or 240,000 depending on the experiment, the AGC off, a 10 (N)CE, and a *m*/*z* 1,000–1010 scan range. The maximum injection time (IT_max_) and multiplex count (MSX count) were adjusted depending on the desired transient length. The time of the scan (*T*_scan_) equals IT_max_ multiplied by the MSX count. For example, a 7 s transient could be achieved by setting IT_max_ to 700 and the MSX count to 10.^26^

### Data Analysis

All data processing and analysis were performed using the Mozaic MSI software (version 2023.2.0.b8, Spectroswiss), as detailed in the Supporting Information. FTMS Booster X2-acquired time-domain data were processed by transient averaging, user-controlled transient truncation, and generation of aFT mass spectra. For each figure, the transient acquisition time equals the longest shown time (either 7 or 9 s) with a shorter time-domain signal achieved through transient truncation using Mozaic MSI software. A half window Kaiser-type apodization and two zero-fills were used prior to the aFT, unless stated otherwise (Figure S2). Thermo RAW data (processed using the standard eFT approach)^27^ were acquired in parallel using the QE HF built-in electronics and also processed in Mozaic software. The mass resolution provided by the RAW (eFT) data is usually 1–10% higher compared with the aFT mass spectra for the same transient length (e.g., 256 ms) (Figure S3). This difference is presumably due to the specifics of the employed apodization functions and their coefficients, which are unknown for the RAW data.

The eFT (RAW data) and aFT (unreduced data) mass spectra were normalized to the base peak (100) and subjected to noise thresholding, peak picking, and recalibration using a reference list for both positive and negative ion mode measurements (Table S2). The mass resolution was calculated using the full-width at half-maximum (fwhm) method with the half-maximum determined starting from the baseline for both eFT and aFT data. All images were normalized to the total ion current (TIC). Lipid annotations were generated by ALEX^123^.^28^ Details about data processing and lipid annotations can be found in the Supporting Information.

## Results and Discussion

### Ultrahigh-Mass Resolution Measurement and Modes of Operation

**Figure 1:**
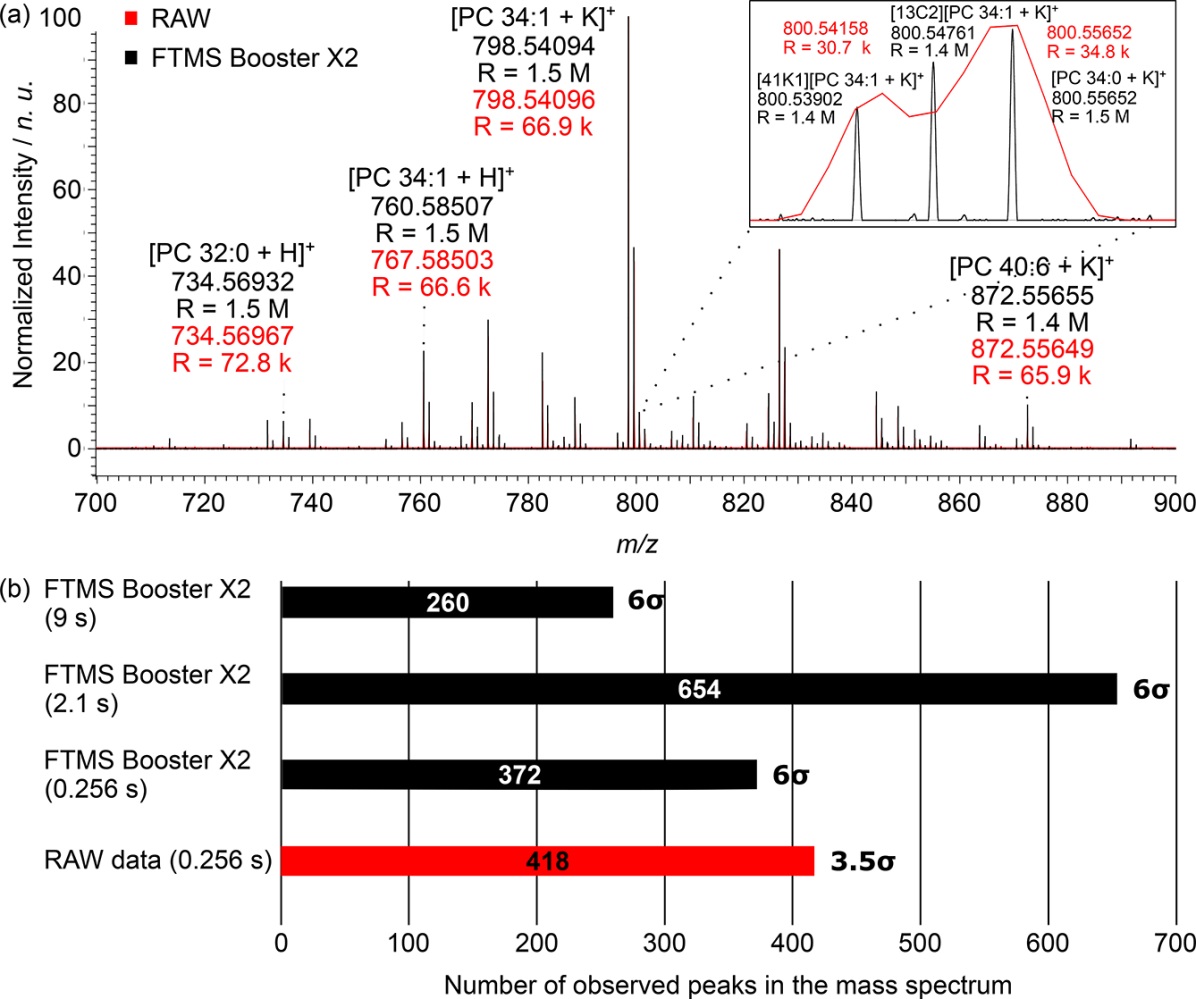
(a) Overlay of single-scan MALDI mass spectra acquired in parallel in a positive ion mode from a mouse brain tissue section coated with a norharmane matrix using FTMS Booster X2 (black, 9 s acquisition time, aFT) and the QE HF RAW data (red, 256 ms acquisition time, eFT). (b) Number of observed peaks in the mass spectrum at different transient lengths. A 6σ noise threshold has been used for all unreduced data (aFT), while a 3.5σ noise threshold has been used for RAW data (eFT). The decrease in the number of peaks for transients above ∼2.1 s is attributed to signal dephasing due to collisions and space charge.^31,32^

Figure 1 shows a positive ion mode single scan mass spectrum for mouse brain tissue, demonstrating a mass resolution of 1,400,000–1,500,000 at *m*/*z* 700–900. At this mass resolution, it should be possible to resolve Na^+^/H^+^ adduct ambiguity (2.35 mDa mass split) or double-bond ambiguity (8.9 mDa mass split), as shown in previous publications with 21 T FT-ICR.^4^ In theory, the mass resolution scales linearly with the transient length and the signal-to-noise (S/N) ratio scales as the square root of the transient length.^29,30^ However, the correlation between the transient length and mass resolution is nonlinear for transient times of above ∼3 s for this setup (Figure S4). The nonlinearity is potentially caused by imperfections in the electrical field and space-charge effects leading to signal dephasing.^31,32^ The achieved mass resolution, as shown in Figure 1, is lower than theoretically expected for a transient length of 9 s (∼2,300,000 for a peak at *m*/*z* 798.5). It is important to note that this finding is consistent with previous work conducted on other FTMS platforms.^15^ The attained mass resolution aligns with the research on FT-ICR-based UHR MSI platforms. Nevertheless, this is the first Orbitrap-based imaging platform that achieved such mass resolution for the MSI of lipids.^4,12,15,16^

The synergy of the extended transients and unreduced (aFT) data representation leads to improved mass accuracy. As a result of subparts-per-million mass accuracy, a higher number of confident sum–composition lipid identifications can be achieved. The elemental composition corresponding to all annotated peaks from Figure 1 is presented in Table S3. As demonstrated, the achieved performance of this UHR platform may remove the double bond ambiguity (DBA) as [13C2] isotopic peaks can be resolved from peaks corresponding to lipid species that differ by one double bond. This is important as DBA is one of the most common causes of misannotation in lipidomics.^33^

Furthermore, S/N is maximized at a transient length of ∼2.1 and then decreases with increasing transient lengths of above ∼2.1 s. Due to space-charge effects and signal dephasing with a longer transient, that is, over ∼2.1 s, there are lesser useful signals and more noise incorporated in a final transient.^32^ This results in a lower number of observed peaks.^32^ The correlation between the S/N and transient length seems to be relatively independent of both *m*/*z* and the abundance of peaks (Figure S5). Moreover, the ion coherence exhibits a correlation with the number of charges present within the orbitrap.^34^ Decreasing the number of charges within the mass analyzer leads to a reduction of ion motion dephasing as well as peak interference and coalescence artifacts.^34^ Notably, the described performance is instrument-specific and relates to the characteristics of the employed Orbitrap and the experimental settings.

These findings allow for two modes of use. First, when UHR is needed to resolve isobaric peaks and generate accurate ion images of an analyte signal in the presence of a nearby isobaric interference, long (e.g., 4–9 s) transients can be used. This mode will allow the resolution of most isobaric signals within the lipid mass range. Second, when higher sensitivity is needed, such as in the case of low-abundance isobars, the transient length could be limited to ∼2 s. Under these conditions, the instrument still provides UHR with a mass resolution of >400,000 at *m*/*z* 800. At the optimal transient length for the S/N, an increase of approximately 1.7 times in S/N for [PC 34:1 + K]^+^ is observed relative to the achieved S/N at a transient length of 512 ms (Figure S4). The required resolution for a given experiment that balances the mass resolution, sensitivity, and throughput will ultimately depend on the experimental requirements and should be determined by the user. The approach described here provides added flexibility in experimental design compared with the standard instrument configuration.

The number of peaks observed (121 averaged scans) between *m*/*z* 650–900 for transient durations of 256 ms (RAW data), 2.1 s (aFT), and 9 s (aFT) was 418, 654, and 260, respectively. Notably, a 3.5σ noise threshold (determined empirically) was used for RAW data to minimize the appearance of the noise peaks in the final mass spectrum, whereas an even stricter noise threshold of 6σ was used for processing the unreduced data (Figure S6). Nevertheless, some noise peaks may have been peak-picked for both the aFT and eFT mass spectra.

The increase in the observed peak count with longer transient (2.1 s) can be attributed to the resolution of numerous isobaric signals, facilitated by increased mass resolution and sensitivity. The reduction in the number of observed peaks for the 9 s transient correlates with the observed decrease in the S/N as the ion coherence diminishes over time. Consequently, the loss of less abundant signals contributes to a reduced peak count, even as some additional isobaric peaks become resolved at a higher mass resolution.

**Figure 2:**
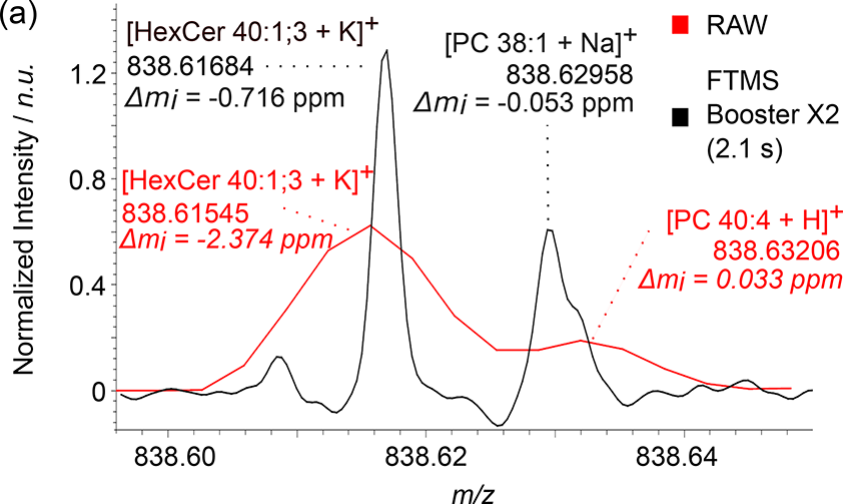
Examples of differences in annotations observed between RAW (eFT) data, shown in red, and aFT data, shown in black, due to the unresolved isobaric features.

The number of lipid annotations was also tracked with the peak count with the 2.1 s transient giving the highest number of annotations and the 9 s transient the lowest. In total, 127, 185, and 77 annotations could be made for the 256 ms (RAW data), 2.1 s (aFT), and 9 s (aFT) data, respectively (see the Supporting Information for the annotation procedure). The list of annotated lipid adducts is shown in Table S4. Notably, some of the annotations found in the RAW data could be assigned as false positives when compared to the 2.1 s transient data, as shown in Figure 2. Misannotated peaks are predominantly either noise features, eFT artifacts, or unresolved isobaric lipid species.^27^ For example, in Figure 2a, the peak at *m*/*z* 838.63206 could potentially be assigned to the [M + H]^+^ ion of PC 40:4 if using a standard mass accuracy tolerance of 2 ppm, whereas the 2.1 s transient reveals an unresolved peak pair with the *m*/*z* of the largest peak instead assigned as the [M + Na]^+^ ion of PC 38:1 (the unresolved right shoulder is likely the [M + H]^+^ ion PC 40:4). Additional examples can be found in Figure S7.

The IFS of [PC 34:1 + K]^+^ up to an M+5 isotopologue can be observed in the mass spectrum from a 2.1 s transient (Figure S8). Alongside the previously mentioned [13C2] isotopic peak and [41K1], it is of significance to highlight the presence of two M+3 isotopologues. One of those signals corresponds to the [13C3] M+3 isotopic peak, while the other M+3 ion signal is the isotopic peak containing one [13C1] and [41K1]. The resolution of IFS confirming the presence of potassium is advantageous for confident lipid identification as it eliminates the possibility of this ion being assigned to [PE O-40:7 + Na]^+^, which could also plausibly be detected in the positive ion mode, and resolving these species would otherwise require a resolution of ∼5,300,000 for both peaks (∼0.19 ppm difference). Given the extreme requirements for mass resolution in such cases, the use of orthogonal techniques such as MS/MS and ion mobility can be advantageous for resolving such isobars. Expectedly, due to the loss of ion coherence with an extended ion detection period (9 s transients), the M+3 and M+4 isotopologues are lost below the S/N threshold despite the mass resolution being ∼3.5 times higher. Similarly, the spectral dynamic range reduces by approximately threefold when the transient duration increases from 2.1 to 9 s. Therefore, a transient length increase above a certain duration adds more noise than the analyte signal. The disappearance of the low abundance peaks also reduces the number of annotations with both monoisotopic and first isotopologue peaks present. For example, the least abundant monoisotopic peak (*m*/*z* 895.68961) that is detected with its M+1 isotopologue and a 2.1 s transient exhibited a relative abundance of 0.13% compared to the abundance of the base peak, that is, a ∼1.5-fold reduction in the spectral dynamic range (single peaks are detected at 0.084%).

### Positive-Mode MALDI MSI of Lipids in a Mouse Brain Tissue Section

**Figure 3:**
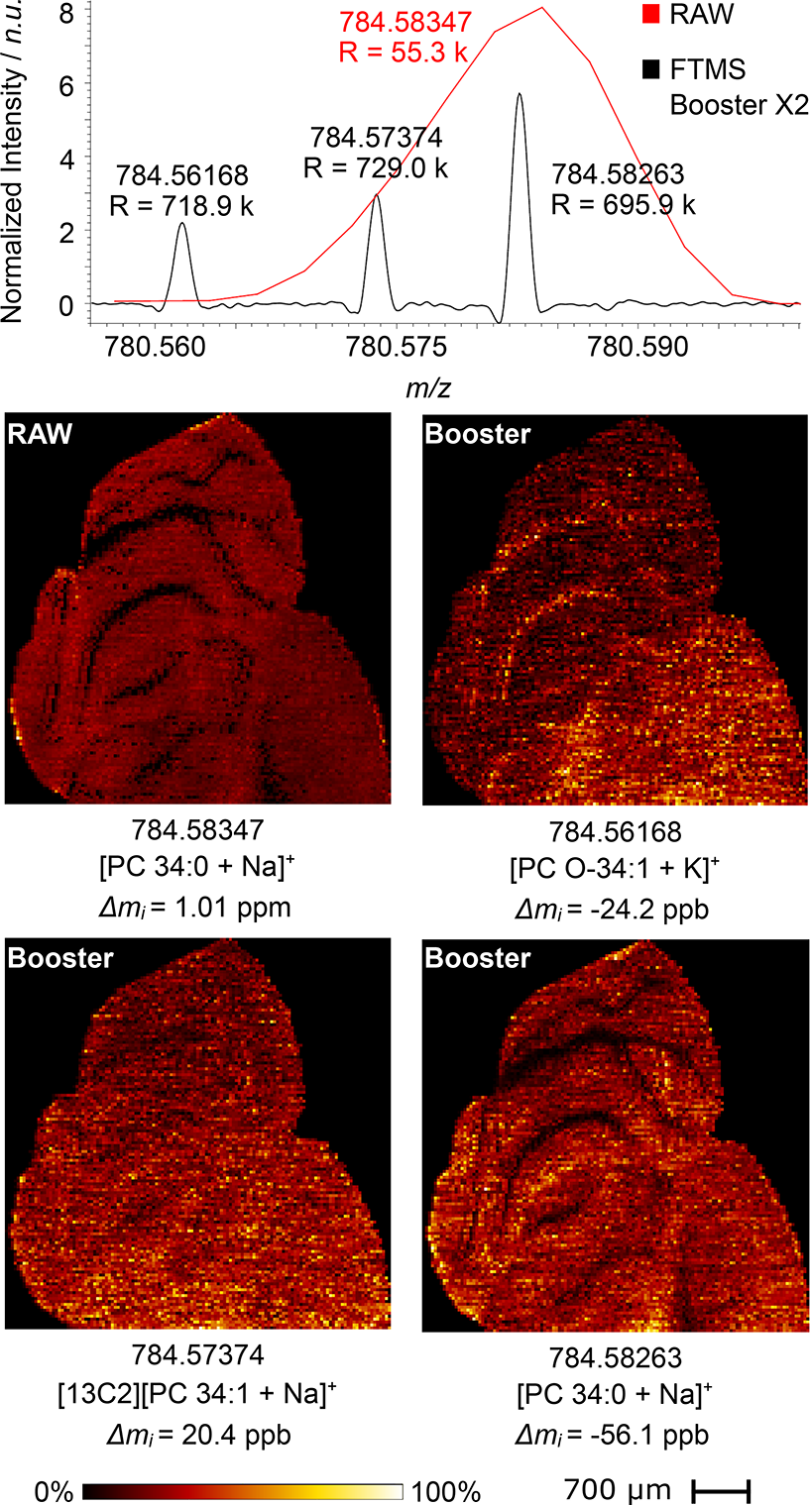
Mouse brain tissue section analyzed in the positive ion mode with the MALDI-enabled QE HF system coupled to the FTMS Booster X2 measured with a pixel size of 70 μm^2^. The Booster-acquired transient length was 7 s, while acquired RAW data corresponds to the 256 ms long transient (120,000 resolution setting of QE HF at *m*/*z* 200). Putative lipid annotations are provided below each brain mass image.

Figure 3 shows an overlay of averaged MALDI mass spectra acquired in parallel from the MALDI-enabled QE HF with the FTMS Booster X2 (7 s transients) and simultaneously with the QE HF electronics (RAW data, 256 ms transients).

The higher mass resolution provided by the FTMS Booster X2 reveals that the RAW peak at *m*/*z* 784.58347 observed with a relative abundance of 5.70% compared to the base peak comprises two unresolved signals at *m*/*z* 784.58263 and 784.57374 (assigned as [PC 34:0 + Na]^+^ and [13C2][PC 34:1 + Na]^+^). We note that, with the maximum 240K resolution of the standard QE HF, these peaks are not baseline-resolved (Figure S9). The increase in sensitivity combined with the resolving peak interference present at lower resolutions, however, enables the detection of a peak at *m*/*z* 784.56168 assigned as [PC O-34:1 + K]^+^. Ion images of the observed lipids [PC 34:0 + Na]^+^, [13C2][PC 34:1 + Na]^+^, and [PC O-34:1 + K]^+^ reveal distinct spatial distributions that would not be discernible using the stock MALDI-enabled QE HF (256 ms long transient, eFT data). [PC 34:0 + Na]^+^ is prominently present within the gray matter of the mouse brain tissue section, exhibiting almost complete absence from the white matter. In contrast, [PC O-34:1 + K]^+^ shows a distribution throughout the tissue with its highest abundance observed in the white matter, contrary to the distribution pattern of [PC 34:0 + Na]^+^. Finally, [13C2][PC 34:1 + Na]^+^ exhibits a relatively uniform distribution across the entire mouse brain tissue section, mirroring the spatial distribution of its monoisotopic peak. This example demonstrates the added biochemical information that can be obtained with the UHR performance and an increase in sensitivity.

An increase in mass accuracy can be observed in the UHR operation mode. This is a consequence of having narrower peaks, whose centroids more accurately represent the exact masses of the corresponding ions. Notably, the standard deviation of observed *m*/*z* values was reduced three times for the unreduced data set compared to the RAW data set (Figure S10). In addition to the improved annotations, resolving the peak interferences also provides more accurate isotope ratios (Figure S11). The presented long transient data perform better in terms of the stability of isotopic peak ratios from pixel to pixel compared to the RAW data. Although the data appear similar for the M+1 isotopic peak, a major improvement can be seen when examining M+2 peaks as that is where DBA occurs.

### Negative-Mode MALDI MSI of a Mouse Brain Tissue Section

**Figure 4:**
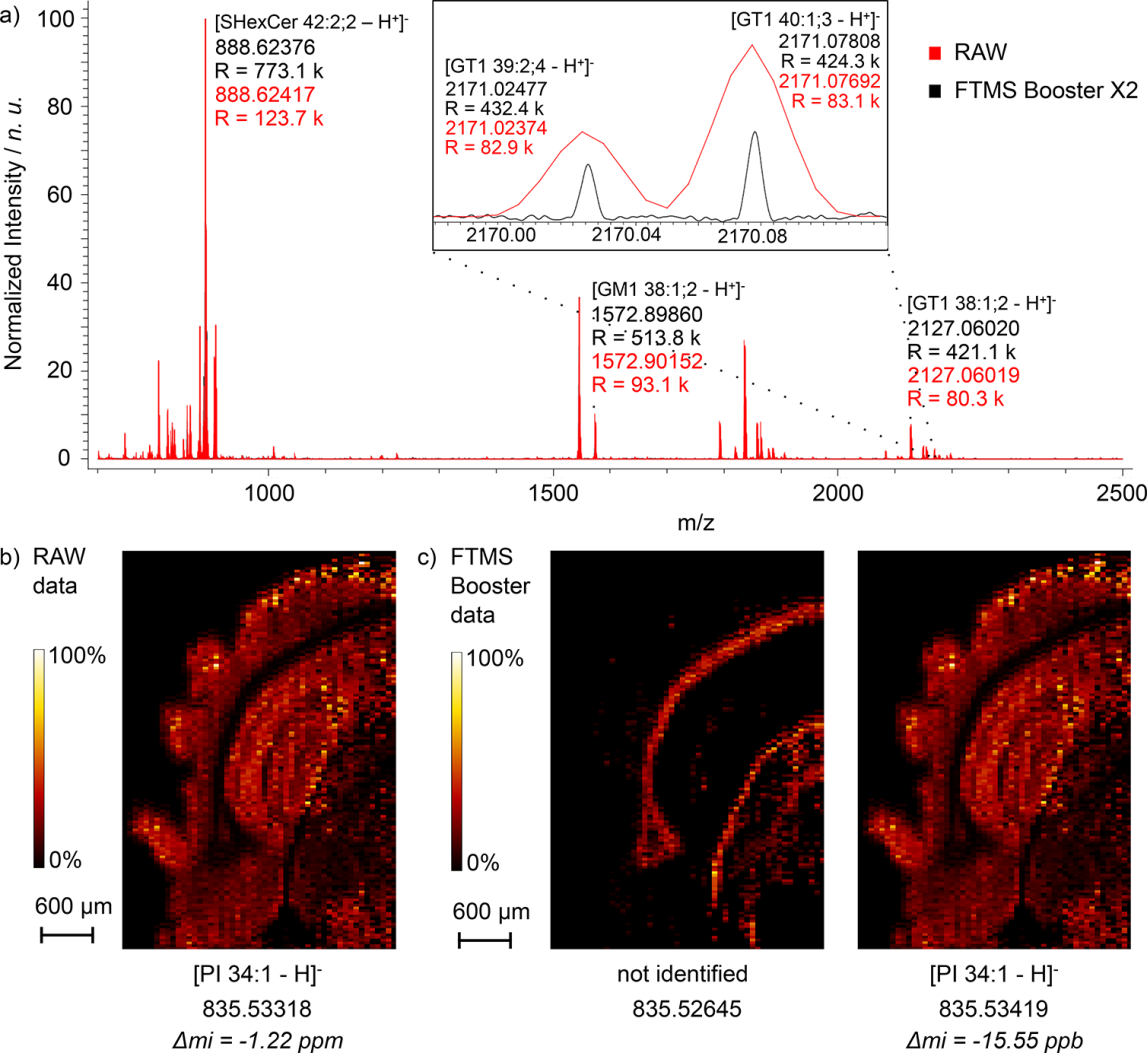
(a) Overlay average mass spectrum acquired with the MALDI-enabled QE HF system coupled to the FTMS Booster X2 measurement of mouse brain tissue in the negative mode. The measurement was performed using the negative mode with a pixel size of 60 μm^2^. Mass spectra were generated from transients of 7 s and 512 ms collected in parallel with the MALDI-enabled QE HF system coupled to the FTMS Booster X2 (black) and the built-in QE HF electronics (red), respectively. (b) Ion image corresponding to the unresolved peak from RAW data at *m*/*z* 835.53318. (c) Ion images of two resolved ion signals from unreduced UHR data at *m*/*z* 835.52645 and *m*/*z* 835.53419.

Typically, an even wider diversity of lipid classes can be detected in the negative ion mode in comparison to positive ion detection. Figure 4a shows the averaged mass spectrum overlay of 7 s transient aFT data (black) along with the 512 ms transient RAW data (red) acquired in the negative ion mode. As mentioned above, the FTMS Booster data show a significant increase in the mass resolving power required to resolve the overlapping ion signals. The number of peaks observed (1000 averaged scans) between *m*/*z* 700–2500 for transient durations of 512 ms (RAW data), 2.1 s (aFT), and 7 s (aFT) are 1550, 2132, and 734, respectively. Similarly to the positive mode data, the 6σ noise threshold was used for all unreduced data, whereas the 3.5σ noise threshold was used for RAW data.

Resolution of the isobaric signal at 835.53318 *m*/*z* results in two distinct ion signals with different spatial distributions in the tissue section (Figure 4b and Figure S12) that are not baseline-resolved in the raw data. The ion signal identified as the [PI 34:1 – H]^−^ at *m*/*z* 835.53419 exhibits an analogous spatial distribution to the ion signal from the RAW data, being localized in the gray matter of the mouse brain section. However, the ion signal at *m*/*z* 835.52645 displays a complementary spatial distribution and is localized exclusively within the white matter of the mouse brain. Sulfatides are one of the most abundant lipid classes detected in negative-ion-mode MALDI-MSI of the brain tissue. The added resolution provides additional confidence in identifying sulfatides at the sum–composition level by baseline resolution of the IFS of the M+2 isotopologues which also confirms the presence of sulfur in the elemental formula (Figure S13).

The mass resolution of Orbitrap mass analyzers is, in theory, inversely proportional to the square root of *m*/*z*, whereas in FT-ICR MS, it is inversely proportional to the *m*/*z*.^35^ This benefits the UHR Orbitrap-based imaging platforms used in certain applications, including native MS and top-down proteomics.^29^Figure 4 demonstrates that resolution levels exceeding 400,000 at approximately *m*/*z* 2,500 are achievable with the acquisition of 7 s long transient signals. This helps the detection of high-mass lipids, such as gangliosides. While we found fewer isobaric signals at a higher *m*/*z* in the negative mode, the subparts-per-million mass accuracy facilitates confident identification of gangliosides such as GT1 38:1;2 (*m*/*z* 2127.06020), GM1 38:1;2 (*m*/*z* 1572.89860), GD1 36:1;2 (*m*/*z* 1835.96480), and GT1 40:1;3 (*m*/*z* 2171.07808).

## Conclusions

The data acquisition and processing enhancements made to the MALDI QE HF MSI platform significantly improved the quality of MALDI imaging in this work. The maximum mass resolution attained at *m*/*z* 800 is ∼1,400,000, representing the highest resolution achieved to date for any Orbitrap-based imaging platform and at the level of work done with FT-ICR-based platforms.^4,12−16^ An increase of 70% in the S/N ratio has been observed for lipid peaks with transient lengths of ∼2 s, which proved to be the optimal length for enhancing instrument sensitivity. Furthermore, the mass accuracy was also improved by an order of magnitude.

These instrumental improvements enable the generation of higher-quality MALDI images that have a more accurate representation of the analyte spatial distribution throughout a sample as more isobaric species are resolved. Crucially, we show that resolved isobaric ions often have different spatial distributions in tissue compared to the ion images obtained from lower-resolution RAW data. Furthermore, higher sensitivity facilitated by the access to unreduced data allowed for the observation of more peaks usually truncated by conventional data reduction. As demonstrated, the higher mass accuracy led to more confident elemental composition identifications. Collectively, these improvements resulted in a 1.5-fold increase in the number of lipid annotations. Overall, the achieved UHR performance of this MALDI MSI platform is of particular benefit for the MSI of lipids due to the complexity of the lipidomics and the large number of isobaric signals typically observed but is also expected to offer notable benefits for other analyte classes.

## Supporting Information Available

Matrix spray application parameters, reference lists used for the recalibration of data for positive and negative modes, elemental composition of peaks annotated in Figure 1, annotations of lipid adducts for different transient lengths for a run in Figure 1, scheme of the developed Orbitrap-based UHR MALDI MSI platform, influence of an apodization function on the mass resolution, overlay of the RAW data mass spectrum and aFT data mass spectrum for the same transient length, correlation of the mass resolution and SNR and transient length from two positive mode measurements, correlation of SNR and transient length for peaks of differing abundance from the same measurement, visual representation of the noise threshold level used for peak picking, examples of possible false positive annotations unique to the RAW data, observation of M+5 [PC 34:1 + K]^+^ isotopologues due to mass resolution and sensitivity improvements with a 2.1 s long transient, overlay of RAW data mass spectrum and aFT data mass spectra showing the resolution of the peak at *m*/*z* 784.58347 at a higher mass resolution, spread of the mass error and dependence of the mass error on the peak intensity after recalibration for RAW data and aFT data, images of isotopic peaks normalized to the intensity of corresponding molecular peaks, overlay of RAW data mass spectrum and aFT data mass spectrum showing the resolution of the isobaric peak at *m*/*z* 835.53318 with a higher mass resolution, and resolution of the M+2 isotopic peak related to the [34S1] simplifies putative identification of sulfatides (PDF)
