# Supplementary material for: Ultrahigh-Mass Resolution Mass Spectrometry Imaging with an Orbitrap Externally Coupled to a High-Performance Data Acquisition System
Source: Anal Chem. 2023 Dec 21;96(2):794–801. doi: 10.1021/acs.analchem.3c04146 (PMC10794996; doi:10.1021/acs.analchem.3c04146)
Supplement: Supplementary file 1 — ac3c04146_si_001.pdf [file ac3c04146_si_001.pdf]

Supporting information:

Ultra-high-mass resolution mass spectrometry imaging with an Orbitrap externally coupled to a high-performance data acquisition system

Andrej Grgic[1], Konstantin O. Nagornov[2], Anton N. Kozhinov[2], Jesse A. Michael[3], Ian G. M. Anthony[1], Yury O. Tsybin[2], Ron M. A. Heeren[1*] and Shane R. Ellis[1,3*]

[1] The Maastricht MultiModal Molecular Imaging (M4I) Institute, Division of Imaging Mass Spectrometry (IMS), Maastricht University, 6229 ER, Maastricht, The Netherlands

[2] Spectroswiss, 1015 Lausanne, Switzerland

[3] Molecular Horizons and School of Chemistry and Molecular Bioscience, University of Wollongong, Wollongong, New South Wales 2522, Australia

*To whom correspondence should be addressed
sellis@uow.edu.au
r.heeren@maastrichtuniversity.nl

Table of Contents

Table S1. Parameters for the automated sprays of norharmane and 2,5-DHA MALDI matrices on mouse brain sections using an HTX M3+ Sprayer. 3

Table S2. (a) Reference list used for recalibration of data acquired in the positive mode MALDI MSI experiments. **(b)** Reference list used for recalibration of data acquired in the negative mode MALDI MSI experiments. 3

Table S3. The elemental composition corresponding to all peaks annotated in Figure 1. 4

Table S4. Annotations and mass accuracies for transient lengths 256 ms (RAW data), 2.1 s (aFT data), and 9 s (aFT data). Parameters used for annotation of mass spectra can be found in Supporting Information.................................. 4

Figure S1. A scheme of the UHR MSI set-up built around a QE HF Orbitrap equipped with the MALDI ion source (Spectroglyph) and coupled to an external data acquisition and processing device (FTMS Booster X2). Mozaic MSI software (Spectroswiss) was used for data processing and data analysis. 6

Figure S2. Apodization function influence on the mass resolution achieved for the [PC 34:1 + K]$^+$ peak at 798.54094 *m/z* with a QE HF.[5] Mass resolution was calculated by doing FT processing on the experimental transient of 9 s by gradually truncating it using the capabilities of Mozaic software. The full width at half maximum (FWHM) method was used to calculate mass resolution and half max was found by measuring from baseline to the peak maximum........................ 7

Figure S3. Overlay of RAW data acquired with the QE HF built-in DAQ/P system and unreduced data acquired with the FTMS Booster X2 at the same transient length (256 ms). 7

Figure S4. (a) Correlation of a transient length and S/N of the [PC 34:1 + K]$^+$ peak at *m/z* 798.54096. **(b)** Correlation of a transient length and mass resolution of the [PC 34:1 + K]$^+$ peak at *m/z* 798.54096. Normalized S/N ratios rather than absolute S/N ratios are plotted to facilitate easier comparison of the two separate imaging experiments.. 8

Figure S5. Correlation of a transient length and S/N of peaks from the same measurement at *m/z* 798.54096, 876.58779, 788.61644, and 742.48648. Normalized S/N ratios rather than absolute S/N ratios are plotted to facilitate the comparison of peaks characterized by the wide range of abundances. 9

Figure S6. Noise thresholding level (red line) used for pick peaking. **(a)** 3.5σ noise threshold for RAW data at 256 ms transient length. **(b)** 6σ noise threshold for unreduced data at 256 ms transient length. **(c)** 6σ noise threshold for unreduced data at 2.1 s transient length. **(d)** 6σ noise threshold for unreduced data at 9 s transient length.. 10

Figure S7. Examples of false positive annotations unique to the RAW (eFT) data. **(a)** A peak in the RAW data was annotated as [PE 34:0 + Na]$^+$ due to the use of a 3 ppm mass accuracy tolerance. In the aFT data this peak is not annotated. Both peaks (RAW data and aFT data) are M+1 [13C1] isotopologues of the peak at 741.53069 *m/z*. **(b)** A peak at 846.46871 *m/z* in the RAW data was annotated as [PS 38:6 + K]$^+$. However, since this peak is absent from the 2.1 s aFT data, is lower than 0.1% of the base peak abundance, and is located near larger peak, it is possible that this is either an eFT artifact or a noise feature leftover after noise thresholding done by the Thermo algorithm. 11

Figure S8. (a) An overlay of MALDI mass spectra acquired in parallel in a positive ion mode from a mouse brain tissue section coated with norharmane matrix using the FTMS Booster X2 (black, 2.1 s acquisition time) and the stock QE HF digitizer to RAW data (red, 256 ms acquisition time) with insets showing enlarged views of the M+2 and M+3 isotopes. At the optimal settings for sensitivty corresponding to a transient length of 2.1 s, IFS is observed up to M+5 isotopologue. **(b)** Zoom-in on the M+4 isotopologue peak. **(c)** Zoom-in on the M+5 isotopologue peak.... ***12***

Figure S9. An overlay of MALDI mass spectra acquired in a positive ion mode using the FTMS Booster X2 (512 ms acquisition time (blue) and 7 s acquisition time (black)) and the stock QE HF digitizer to RAW data (red, 256 ms acquisition time). 13

Figure S10. (a) Spread of mass errors observed for a peak at 798.54097 *m/z* in unreduced data with a transient length of 7 seconds. **(b)** Spread of mass errors observed for a peak at 798.54095 *m/z* in RAW data with a transient length of 256 ms. **(c)** Mass error vs. Intensity plot in unreduced data with transient length of 7 seconds **(d)** Mass error vs. Intensity plot in RAW data with transient length of 256 ms. Both RAW data and aFT data have been recalibrated using the reference list provided in Table S2a.. 14

Figure S11. Images of [PC 34:1 + K]$^+$ lipid peak and corresponding isotopic peaks normalized to the intensity of the molecular peak at 798.54094 *m/z* for unreduced data and 798.54088 *m/z* for RAW data. **(a)** Images generated with the FTMS Booster X2 acquired data set. **(b)** Images generated with the RAW (eFT) data set collected by the in-built DAQ unit of the Orbitrap QE HF. As the peak at 800.5 *m/z* remains unresolved, the generated image represents the combined spatial distribution of [41K][PC 34:1 + K]$^+$, [13C2][PC 34:1 + K]$^+$, and [PC 34:0 + K]$^+$.. 15

Figure S12. An overlay of MALDI mass spectra acquired in a negative ion mode using the FTMS Booster X2 (black, 7 s acquisition time) and the stock QE HF digitizer to RAW data (red, 512 ms acquisition time). As shown in Figure 4, resolution of the isobaric peak at *m/z* 835.53318 results in two distinct ion signals with different spatial distributions... 16

Figure S13. Mass spectrum of SHexCer 42:2;2 and corresponding M+2 isotopic peaks. **(a)** The molecular peak of [SHexCer 42:2;2 - H+]- **(b)** Overlay of acquired mass spectra with a simulated isotopic profile in FTMS Simulator (Mozaic software).[7] 16

References 17

Additional details about the MALDI MSI. The time of the run is calculated with the following equation:

$$t_{run} = \frac{N\ (number\ of\ pixels)}{2} \times\ (IT_{max}\ (AIF\ mode) +\ T_{scan}\ (tSIM\ mode))$$

The obtained t_{run} value is then used as a run time in Thermo Xcalibur Instrument Setup software, after which the new method is saved. The method is selected in a QE HF acquisition tab. Prior to the start of a run, the QE HF is put into a stand-by mode so it stops generating time-domain data. First, the single acquisition mode run is set into the signal waiting mode on the FTMS Booster X2 in the Booster CTRL software (Spectroswiss). Subsequently, the run is started on the QE in the Tune software. Finally, the ion source control software starts a MALDI MSI run. Due to the contact closure signal that is sent by the MALDI ion source, no transients will be acquired until the run is started on the MALDI source. This sequence is necessary to keep all components of this instrument set-up synchronized.
Time per pixel for conducted experiments:

- Run 1 – 9550 ms per pixel (analytical scan + dummy scan) – 310 pixels – 49.3 min
- Run 2 – 7550 ms per pixel (analytical scan + dummy scan) – 12320 pixels – 1550 min – 25.8 h
- Run 3 – 7550 ms per pixel (analytical scan + dummy scan) – 12680 pixels – 1595 min – 26.6 h

One of the main considerations when planning a MALDI-enabled Orbitrap MSI experiment in the UHR mode is the total acquisition (experimental) time. Depending on the ion injection (accumulation) time, the Orbitrap QE HF can record almost two scans per second when it is set to the highest resolution setting (240,000 at *m/z* 200) or more when it is set to the lower resolution settings. However, the time of MALDI-MSI experiments increases proportionally with the transient length. This means that the experiment time will increase 14-fold for 7 s long transient acquisitions. Measurement time could be shortened in multiple ways. First, being more selective with an area on- and/or off-tissue that is measured will reduce the total number of pixels. Second, lowering the spatial resolution of measurements reduces total number of pixels.

Additionally, the acquisition of unreduced data translates into much larger imaging data sets. Experiments on the MALDI-enabled QE HF system coupled to the FTMS Booster X2 generate approximately 1 GB of data for 1 minute of acquisition. This, in combination with a longer run time, results in an increase in data size from 1 GB or less for RAW data to hundreds of GB for unreduced data. To cope with this large file size, further improvements to the data acquisition software are needed. For example, transients can be recorded starting from a higher *m/z* (lower frequency) value to proportionally (and significantly) reduce the number of data points per transient.

In addition to the aforementioned conventional methods, computational approaches can also reduce measurement time. For example, a 2-4-fold reduction in transient length can be offered by performing super-resolution signal processing, e.g., least squares fitting, instead of the FT.[1] Another potential approach is the utilization of compressed sensing with subspace imaging-based sparse sampling to enhance throughput.[2] In this technique, a high-resolution

dataset acquired from a small area is used to reconstruct low-resolution data collected from a larger area, such as an entire section. Furthermore, randomly sampling portions of a pixel, rather than the whole pixel, offers additional opportunities for the reduction of measurement time.[2] Alternatively, rather than focusing on reducing measurement time, more efficient use of time spent during recording long transient can be considered. For example, externally coupling FTMS Booster to instruments that can perform simultaneous acquisition of MS/MS in a parallel analyzer, while the long transient is being recorded.[3]

Apodization function. When generating an aFT mass spectrum, an apodization function is applied to the transient.[4,5] The half window Kaiser-type apodization function is utilized for generating all aFT mass spectra presented in this paper due to its advantages, such as the absence of negative side lobes. However, while the half-window Kaiser-type apodization function is preferred in absorption mode, the full-window Kaiser-type apodization function produces even narrower peaks, which can be advantageous. Figure S2 demonstrates the mass resolution achieved for the $[PC\ 34:1 + K^+]^+$ peak at *m/z* 798.54094 with full-window Kaiser-type mFT, full-window Kaiser-type aFT, and half-window Kaiser-type aFT.

Data recalibration. For the images shown in this paper, the data have been recalibrated on a pixel-by-pixel basis using one reference list for the positive mode and one reference list for the negative mode. Reference lists used can be seen in Table S3.

Determining the σ value used for noise thresholding. The logarithmic method was used for the estimation of the standard deviation of noise and for the noise thresholding as described in the paper by Zhurov et al.[6] The number of used bins is 100. The noise threshold has been set to 6σ for aFT data and 3.5σ for RAW data. A graphical representation of peak picking can be seen in Figure S6.

Annotation of lipids. Lipid annotations were generated by ALEX[123].[3] Averaged profile-mode spectral data in text format from RAW and aFT files in positive ion mode was processed with the ALEX Xtractor module (0.005 and 0.001 Da search tolerance, respectively) using a target list containing a range of species within Cer, CerP, HexCer, Hex2Cer, SHexCer, SM, DAG, TAG, PA, PI, PS, PG, PC, PE, PC-O and PE-O lipid classes as multiple adducts. Annotations were subjected to an intensity threshold (as described in Figure S2)[3], mass accuracy threshold (0.5 ppm for aFT files, 2.5 ppm for RAW files, determined by evaluating mass errors of common lipids), and requirement for observation of potassium adducts (Tableau Professional Desktop Edition 2022.2.14). Annotations were manually curated by considering expected common species and ionization behavior for each class. Annotations for PE species in positive ion mode are shared with isomeric odd-chain PC species as this MS^1-based analysis cannot differentiate isomers. Note there are slight discrepancies in the mass errors reported by ALEX[123] and Thermo or Spectroswiss software as ALEX[123] uses the highest data point across a peak rather than the centroided value.

Table S1. Parameters for the automated sprays of norharmane and 2,5-DHA MALDI matrices on mouse brain sections using an HTX M3+ Sprayer.

	**Positive mode measurements**	**Negative mode measurements**
**Matrix**	Norharmane	2,5-DHA + $(NH_4)_2SO_4$
**Solvent**	2:1 $CHCl_3$:MeOH	70% EtOH
**Nozzle Temperature / *°C***	30	80
**Flow Rate / *µL min^{-1}***	120	30
**Nozzle Velocity / *mm min^{-1}***	1200	1200
**Matrix Concentration / *mg mL^{-1}***	7	5
**Number of Passes**	12	20
**Spray Pattern**	criss-cross (CC)	criss-cross (CC)
**Track spacing / *mm***	3	2
**Drying time / *s***	30	30
**N_2 gas pressure / *psi***	10	10
**Nozzle height / *mm***	40	40

Table S2. (**a**) Reference list used for recalibration of data acquired in the positive mode MALDI MSI experiments. (**b**) Reference list used for recalibration of data acquired in the negative mode MALDI MSI experiments.

(a)

Positive mode recalibration		
Lipid	Elemental composition	*m/z*
$[PC\ 34:1 + H]^+$	C42H83NO8P	760.585082
$[PC\ 34:1 + Na]^+$	C42H82NO8PNa	782.567024
$[PC\ 36:1 + K]^+$	C44H86NO8P^{39}K	826.572264
$[PC\ 38:6 + K]^+$	C46H80NO8P^{39}K	844.525313

(b)

Negative mode recalibration		
Lipid	Elemental composition	*m/z*
$[SHexCer\ 40:1;2 - H]^-$	C46H88NO11S	862.608357
$[PI\ 38:4 - H]^-$	C47H82O13P	885.549853
$[SHexCer\ 42:1;3 - H]^-$	C48H92NO12S	906.634572
$[GM1\ 36:1;2 -H]^-$	C73H130N3O31	1544.869378

Table S3. The elemental composition corresponding to all peaks annotated in Figure 1.

Elemental composition	m/z_{exp}	m/z_{theor}	Δm_i / *ppm*
C40H81NO8P	734.56932	734.56943	-0.152
C42H83NO8P	760.58507	760.58508	-0.013
C42H82NO8P^{39}K	798.54094	798.54096	-0.025
C42H82O8^{41}NP^{39}K	800.53902	800.53908	-0.075
C40^{13}C2H82O8NP^{39}K	800.54761	800.54767	-0.075
C42H84O8NP^{39}K	800.55652	800.55661	-0.112
C48H84O8NP^{39}K	872.55655	872.55661	-0.069

Table S4. Annotations and mass accuracies expressed in ppm for transient lengths 256 ms (RAW data), 2.1 s (aFT data), and 9 s (aFT data). Parameters used for annotation of mass spectra can be found in Supporting Information.

			RAW data (256 ms)			aFT data (2.1 s)			aFT data (9 s)		
			Assigned adducts (ppm error)								
Lipid class	Elemental composition	Lipid species	+H+	+K+	+Na+	+H+	+K+	+Na+	+H+	+K+	+Na+
DAG	C39H74O5	DAG 36:1		1.388			-0.216				
HexCer	C38H73NO9	HexCer 32:1;3					-0.032				
	C42H81NO8	HexCer 36:1;2					-0.076				
	C42H81NO9	HexCer 36:1;3					-0.059				
	C46H87NO8	HexCer 40:2;2					-0.095				
	C46H87NO9	HexCer 40:2;3					-0.061				
	C46H89NO8	HexCer 40:1;2					0.292				
	C46H89NO9	HexCer 40:1;3		-1.450	-2.412		0.093	0.033		-0.128	
	C46H91NO7	HexCer 40:0;1		1.610							
	C46H91NO9	HexCer 40:0;3					-0.353				
	C48H91NO8	HexCer 42:2;2		-1.297	-0.863		-0.203	-0.053		-0.148	-0.104
	C48H91NO9	HexCer 42:2;3		1.461	1.355		0.083	0.250		-0.168	
	C48H93NO8	HexCer 42:1;2					-0.031				
	C48H93NO9	HexCer 42:1;3		-1.096	-0.028		-0.059	0.043		-0.194	
PC	C34H66NO8P	PC 26:1					0.358				
	C34H68NO8P	PC 26:0				0.194	0.122				
	C38H74NO8P	PC 30:1					-0.488				
	C38H76NO8P	PC 30:0	0.490	0.645		-0.082	-0.095				
	C40H78NO8P	PC 32:1	-0.493	-1.878	0.199	-0.149	-0.055	-0.053	-0.137	-0.083	
	C40H80NO8P	PC 32:0	0.001	-1.049	-0.233	0.042	0.157	-0.026	-0.136	0.017	-0.123
	C42H78NO8P	PC 34:3					-0.016				
	C42H80NO8P	PC 34:2	-0.571	0.631	1.088	-0.003	0.202	0.047		-0.070	-0.214
	C42H82NO8P	PC 34:1	1.782	1.509	-0.092	0.137	0.091	0.229	-0.013	0.044	0.022
	C42H84NO8P	PC 34:0		0.566		-0.220	0.041	0.066	-0.139	-0.120	-0.141
	C44H78NO8P	PC 36:5					-0.049				
	C44H80NO8P	PC 36:4		1.336	0.286		0.091	-0.085	-0.146	-0.079	-0.191
	C44H82NO8P	PC 36:3		-1.610	0.939		0.115			-0.169	
	C44H84NO8P	PC 36:2	1.758	0.602	-0.479	0.024	-0.029	-0.137	-0.074	-0.085	-0.074
	C44H86NO8P	PC 36:1	0.209	0.217	-0.210	-0.109	0.059	-0.028	-0.039	0.005	-0.023
	C44H88NO8P	PC 36:0					-0.133				
	C46H78NO8P	PC 38:7		-1.892			-0.068				
	C46H80NO8P	PC 38:6	-2.047	-1.879	-1.232	0.171	-0.143	-0.251	-0.105	-0.011	-0.132
	C46H82NO8P	PC 38:5		-1.121			-0.044	-0.017		-0.105	-0.070

	C46H84NO8P	PC 38:4		0.334	-0.387		0.181	-0.171	-0.262	-0.057	-0.107
	C46H86NO8P	PC 38:3				0.018	0.014			-0.051	
	C46H88NO8P	PC 38:2	-1.607	1.220		-0.047	-0.130	-0.011	-0.044	-0.137	-0.233
	C46H90NO8P	PC 38:1	-0.421	0.545		-0.062	0.167	-0.266	-0.345	-0.075	-0.198
	C48H80NO8P	PC 40:8					0.003				
	C48H82NO8P	PC 40:7		-0.975	0.140		-0.055	-0.110	-0.287	-0.075	-0.177
	C48H84NO8P	PC 40:6	0.688	0.406	-0.188	0.046	-0.097	-0.037	-0.064	-0.058	-0.106
	C48H86NO8P	PC 40:5					0.286	-0.089		0.043	
	C48H88NO8P	PC 40:4	-0.073	-0.695	0.455		0.133	0.158		-0.050	
	C48H90NO8P	PC 40:3					-0.142				
	C48H92NO8P	PC 40:2	-1.158	-1.867		0.080	-0.111	-0.062		-0.118	
	C48H94NO8P	PC 40:1	-0.491	-0.600		-0.272	0.182	-0.152	-0.215	-0.062	
	C50H86NO8P	PC 42:7	-2.343	0.132		-0.294	0.326	-0.252			
PC O-	C40H80NO7P	PC O-32:1				-0.096	-0.024				
	C40H82NO7P	PC O-32:0				-0.219	-0.022				
	C42H82NO7P	PC O-34:2				0.424	0.179				
	C42H84NO7P	PC O-34:1	1.034	-2.169	0.912	0.111	-0.087	-0.217	-0.158	-0.071	
	C42H86NO7P	PC O-34:0					0.137				
	C44H82NO7P	PC O-36:4					0.083				
	C44H84NO7P	PC O-36:3					-0.127				
	C44H86NO7P	PC O-36:2				-0.227	-0.116				
	C44H88NO7P	PC O-36:1				0.010	0.062	-0.075			
	C46H80NO7P	PC O-38:7		0.905	-0.223						
	C46H82NO7P	PC O-38:6					-0.122	0.446			
	C46H84NO7P	PC O-38:5					-0.368				
	C46H90NO7P	PC O-38:2					-0.272				
PE	C33H64NO8P	PE 28:1					0.025				
	C39H76NO8P	PE 34:1		-1.934		-0.071	-0.337				
	C39H78NO8P	PE 34:0	1.035	0.597	-0.628	0.024	-0.086				
	C41H78NO8P	PE 36:2		1.873			0.128				
	C41H80NO8P	PE 36:1	2.094	-0.905		0.319	0.131			-0.190	
	C41H82NO8P	PE 36:0				0.250	0.177				
	C43H74NO8P	PE 38:6		0.669			0.055	0.411		-0.106	
	C43H76NO8P	PE 38:5		-0.747			-0.036				
	C43H78NO8P	PE 38:4		-0.363			-0.243	0.022			
	C43H82NO8P	PE 38:2		-2.046		-0.273	0.132				
	C43H84NO8P	PE 38:1	0.081	-0.409	0.593	-0.221	0.142	-0.010	-0.141	-0.197	
	C43H86NO8P	PE 38:0				-0.255	0.260				
	C45H76NO8P	PE 40:7					-0.099	0.412			
	C45H78NO8P	PE 40:6		0.017	-2.130		-0.117	-0.093		-0.169	0.025
	C45H82NO8P	PE 40:4				0.128	0.018				
	C45H86NO8P	PE 40:2				0.082	0.026				
	C45H88NO8P	PE 40:1	-0.689	0.021	0.411	-0.138	-0.149				
	C47H82NO8P	PE 42:6	1.545	-0.113		0.124	-0.319				
	C47H92NO8P	PE 42:1				-0.219	0.351				
	C49H96NO8P	PE 44:1					0.413				
PE O-	C39H76NO7P	PE O-34:2	-0.941	1.084		0.021	-0.226				
	C41H78NO7P	PE O-36:3	0.749	-1.640		0.049	0.112	0.168	-0.099	-0.082	
	C41H80NO7P	PE O-36:2	0.457	1.129		0.148	-0.100			-0.018	
	C41H82NO7P	PE O-36:1		-0.666							
	C43H74NO7P	PE O-38:7		0.304	-1.681		-0.287	0.143			
	C43H76NO7P	PE O-38:6		-1.819	-0.852						
	C43H78NO7P	PE O-38:5		-1.680			0.014				
	C43H80NO7P	PE O-38:4					-0.019				
	C43H82NO7P	PE O-38:3		-2.396			0.190				
	C43H84NO7P	PE O-38:2					0.132			-0.084	
	C45H76NO7P	PE O-40:8					0.234	0.393			
	C45H78NO7P	PE O-40:7		0.640	1.698		0.088	0.281		-0.194	0.233
	C45H82NO7P	PE O-40:5		-0.417			-0.098				
PS	C42H80NO10P	PS 36:1					0.189				
	C42H82NO10P	PS 36:0		-2.217			0.264				
	C44H74NO10P	PS 38:6		-1.067							
	C46H78NO10P	PS 40:6		-2.286			-0.255				
SM	C39H79N2O6P	SM 34:1;2	-2.037	0.953		-0.264	0.046				
	C41H81N2O6P	SM 36:2;2	-0.835	-0.962		-0.042	0.052	-0.043	-0.299	-0.033	-0.363

	C41H83N2O6P	SM 36:1;2	1.098	-0.344	0.072	-0.145	-0.195	0.045	-0.078	-0.056	-0.165
	C41H85N2O6P	SM 36:0;2					0.084				
	C43H85N2O6P	SM 38:2;2					0.170				
	C43H87N2O6P	SM 38:1;2	1.423	-1.686	-0.842	-0.240	0.068	0.155	-0.170	-0.149	
	C45H89N2O6P	SM 40:2;2		-1.532		0.034	0.217				
	C45H91N2O6P	SM 40:1;2		-2.273		-0.234	-0.225				
	C47H93N2O6P	SM 42:2;2	-1.914	0.596	0.827	0.160	-0.119	-0.205	-0.122	-0.067	-0.255
	C47H95N2O6P	SM 42:1;2		-0.093		-0.055	-0.019			-0.086	
			29	63	29	46	101	40	21	39	20
		Sum lipid species annotations	63			101			40		
		Sum adduct annotations	121			187			80		

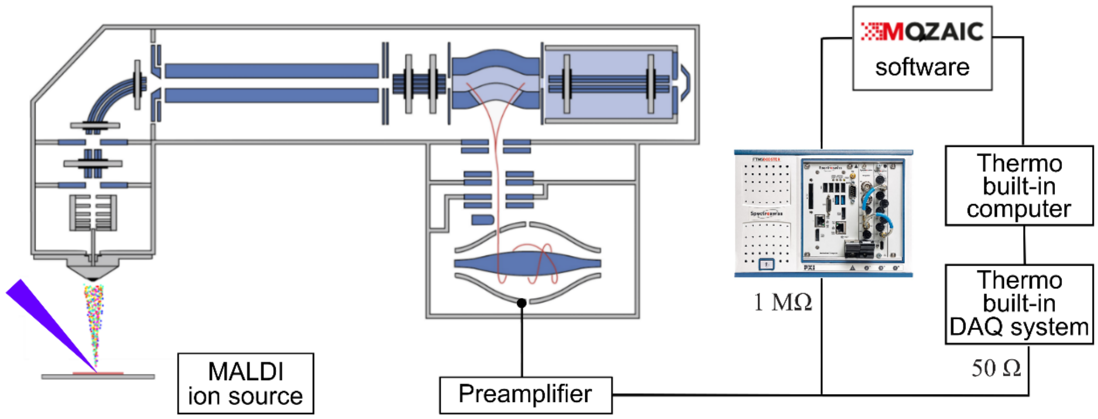

Figure S1. A scheme of the UHR MSI set-up built around a QE HF Orbitrap equipped with the MALDI ion source (Spectroglyph) and coupled to an external data acquisition and processing device (FTMS Booster X2). Mozaic MSI software (Spectroswiss) was used for data processing and data analysis.

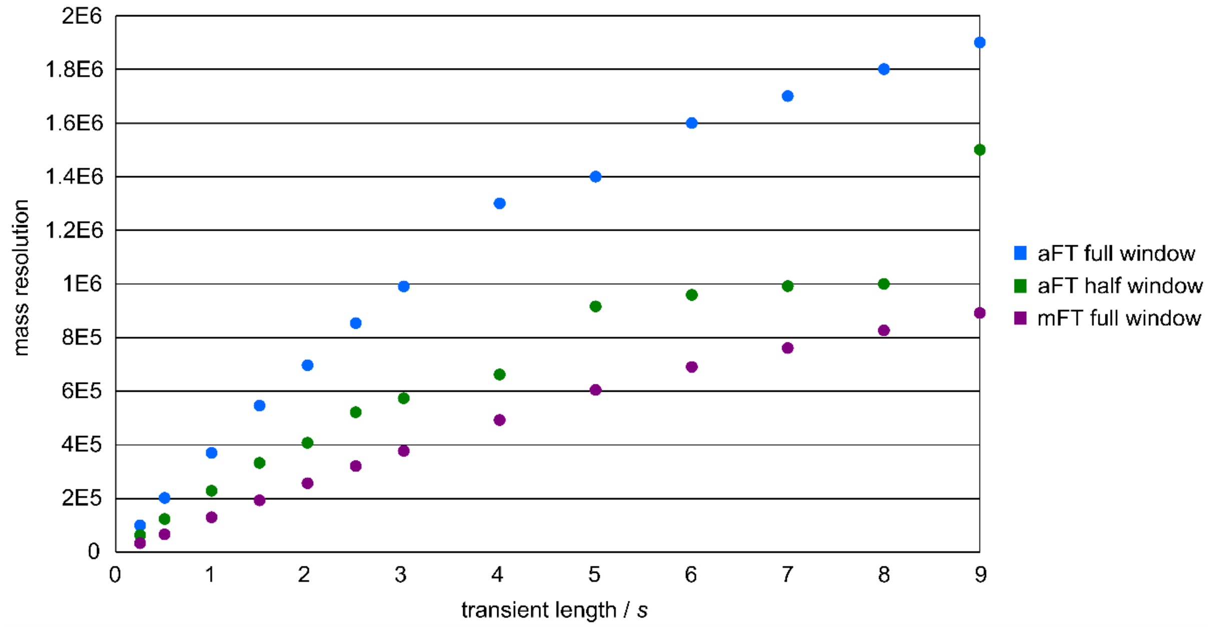

Figure S2. Apodization function influence on the mass resolution achieved for the [PC 34:1 + K]+ peak at 798.54094 *m/z* with a QE HF.[5] Mass resolution was calculated by doing FT processing on the experimental transient of 9 s by gradually truncating it using the capabilities of Mozaic software. The full width at half maximum (FWHM) method was used to calculate mass resolution and half max was found by measuring from baseline to the peak maximum.

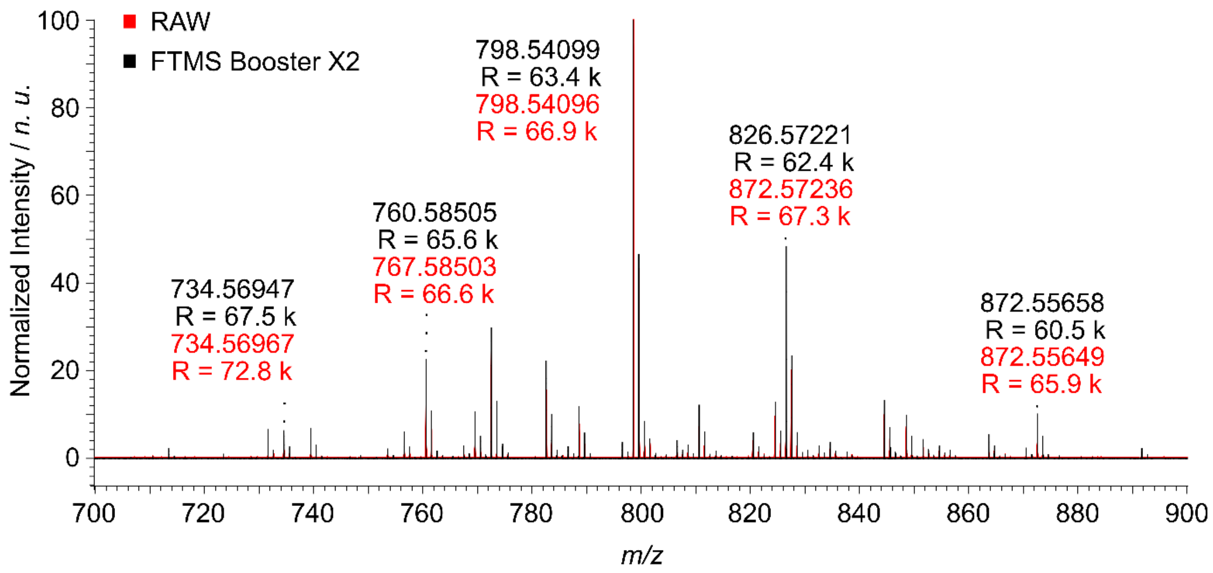

Figure S3. Overlay of RAW data acquired with the QE HF built-in DAQ/P system and unreduced data acquired with the FTMS Booster X2 at the same transient length (256 ms).

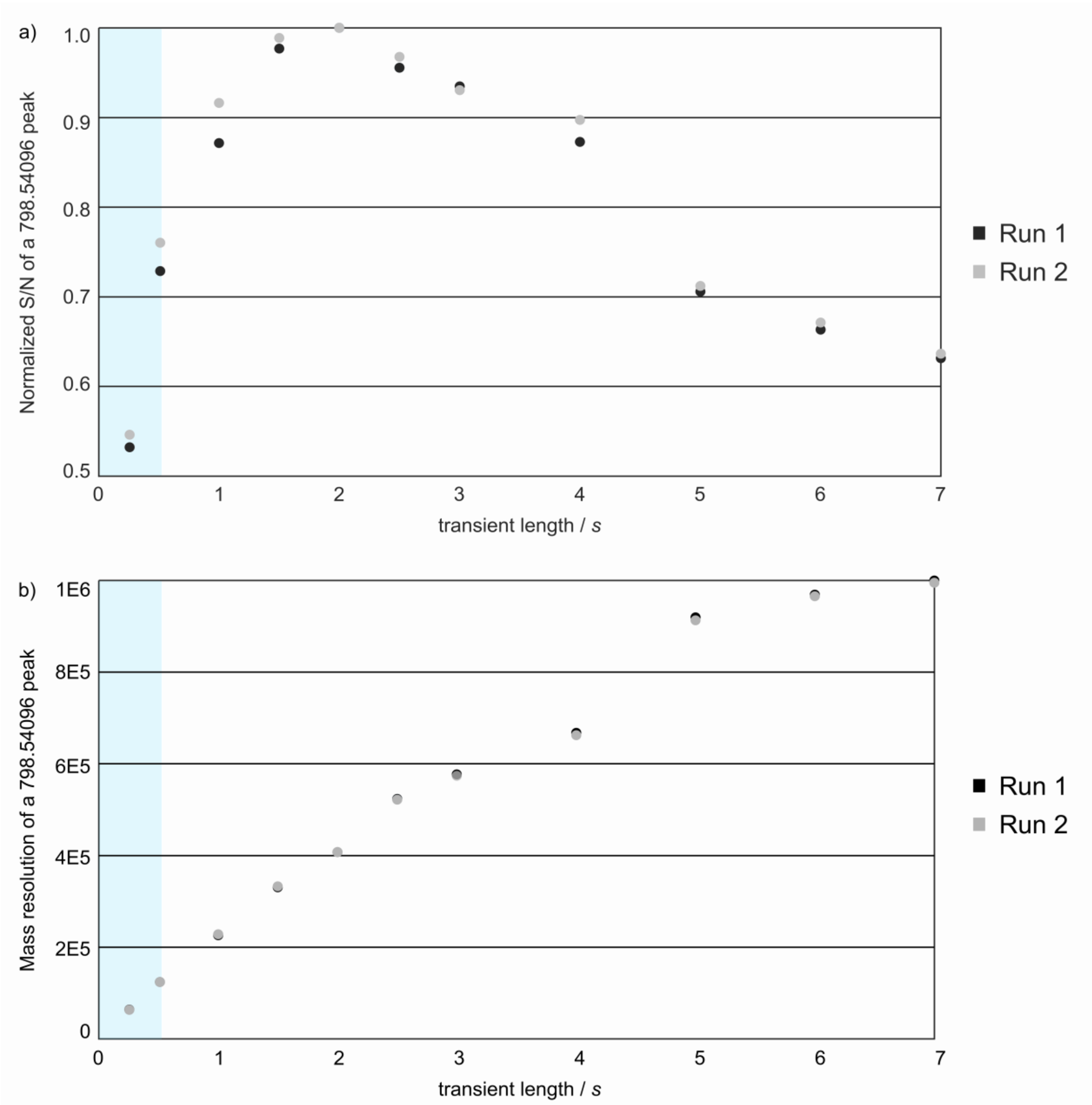

Figure S4. (**a**) Correlation of a transient length and S/N of the [PC 34:1 + K]$^+$ peak at *m/z* 798.54096. (**b**) Correlation of a transient length and mass resolution of the [PC 34:1 + K]$^+$ peak at *m/z* 798.54096. Normalized S/N ratios rather than absolute S/N ratios are plotted to facilitate easier comparison of the two separate imaging experiments.

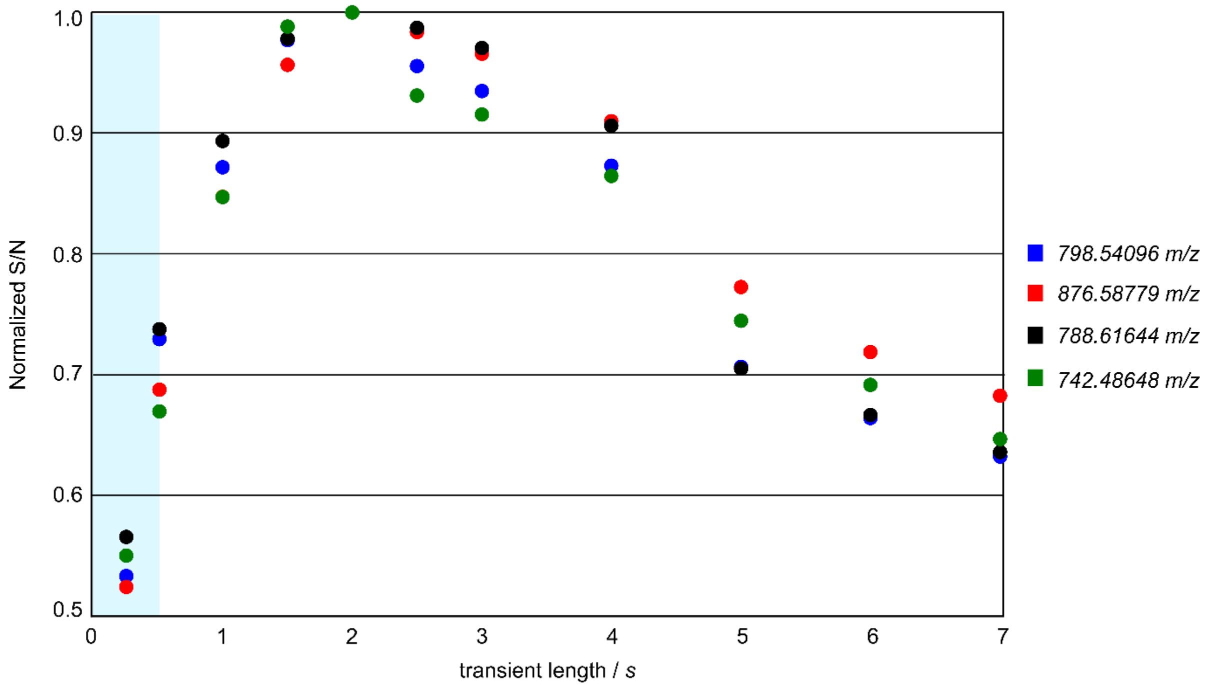

Figure S5. Correlation of a transient length and S/N of peaks from the same measurement at *m/z* 798.54096, 876.58779, 788.61644, and 742.48648. Normalized S/N ratios rather than absolute S/N ratios are plotted to facilitate the comparison of peaks characterized by the wide range of abundances.

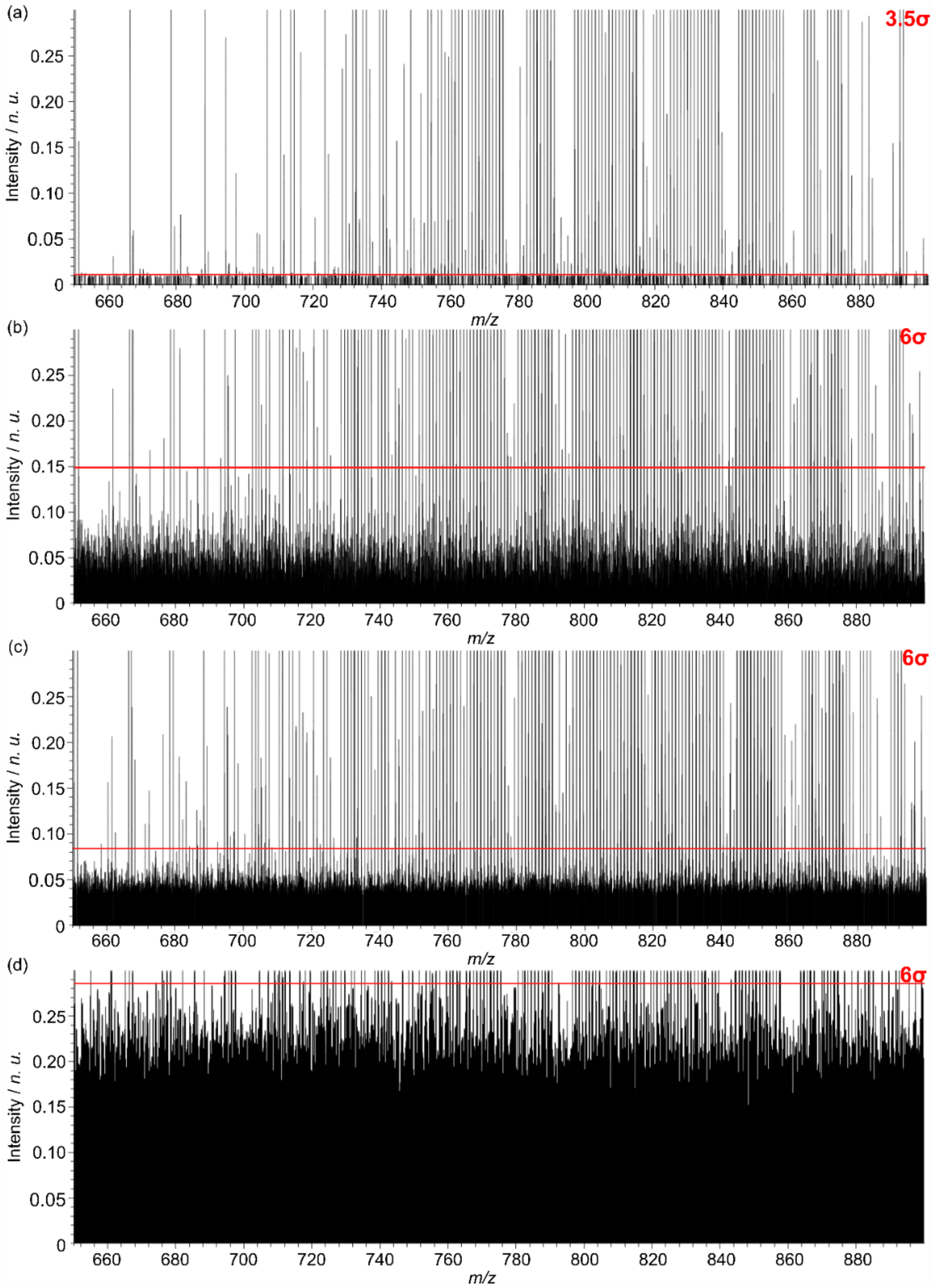

Figure S6. Noise thresholding level (red line) used for pick peaking. (**a**) 3.5σ noise threshold for RAW data at 256 ms transient length. (**b**) 6σ noise threshold for unreduced data at 256 ms transient length. (**c**) 6σ noise threshold for unreduced data at 2.1 s transient length. (**d**) 6σ noise threshold for unreduced data at 9 s transient length.

The 6σ noise threshold was applied to all unreduced data, as it is considered a gold standard for peak picking. However, in the case of RAW data, a noise threshold of 3.5σ was utilized. This was because RAW data had been preprocessed by the Thermo algorithm, including peak picking. The value of 3.5σ was determined empirically to find a balance: to prevent the exclusion of real peaks while simultaneously minimizing the inclusion of noisy peaks.

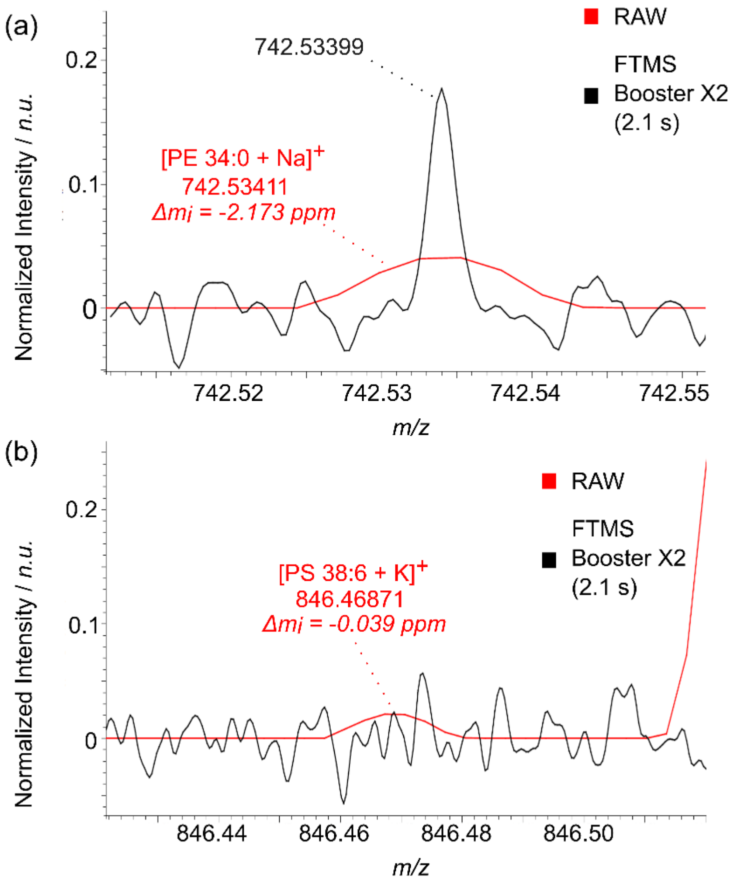

Figure S7. Examples of false positive annotations unique to the RAW (eFT) data. **(a)** A peak in the RAW data was annotated as $[PE\ 34{:}0 + Na]^+$ due to the use of a 3 ppm mass accuracy tolerance. In the aFT data this peak is not annotated. Both peaks (RAW data and aFT data) are M+1 [13C1] isotopologues of the peak at 741.53069 *m/z*. **(b)** A peak at 846.46871 *m/z* in the RAW data was annotated as $[PS\ 38{:}6 + K]^+$. However, since this peak is absent from the 2.1 s aFT data, is lower than 0.1% of the base peak abundance, and is located near larger peak, it is possible that this is either an eFT artifact or a noise feature leftover after noise thresholding done by the Thermo algorithm.

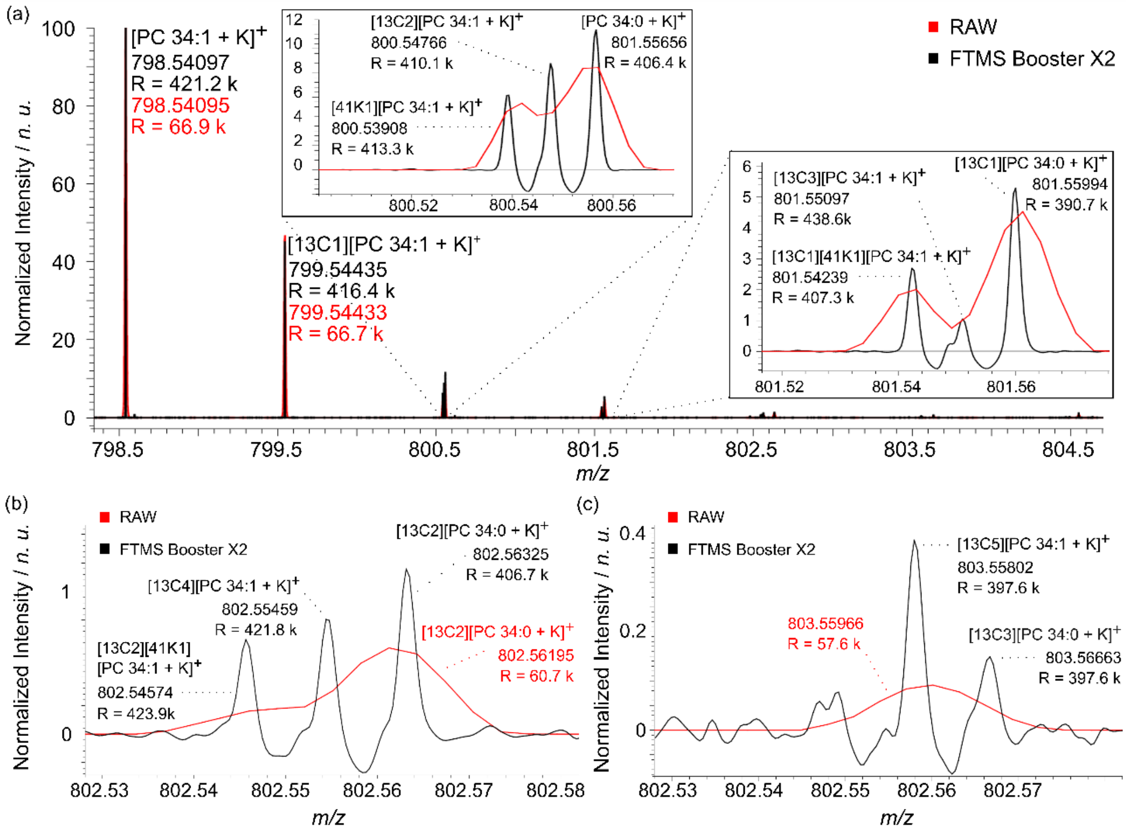

Figure S8. (**a**) An overlay of MALDI mass spectra acquired in parallel in a positive ion mode from a mouse brain tissue section coated with norharmane matrix using the FTMS Booster X2 (black, 2.1 s acquisition time) and the stock QE HF digitizer to RAW data (red, 256 ms acquisition time) with insets showing enlarged views of the M+2 and M+3 isotopes. At the optimal settings for sensitivty corresponding to a transient length of 2.1 s, IFS is observed up to M+5 isotopologue. (**b**) Zoom-in on the M+4 isotopologue peak. (**c**) Zoom-in on the M+5 isotopologue peak.

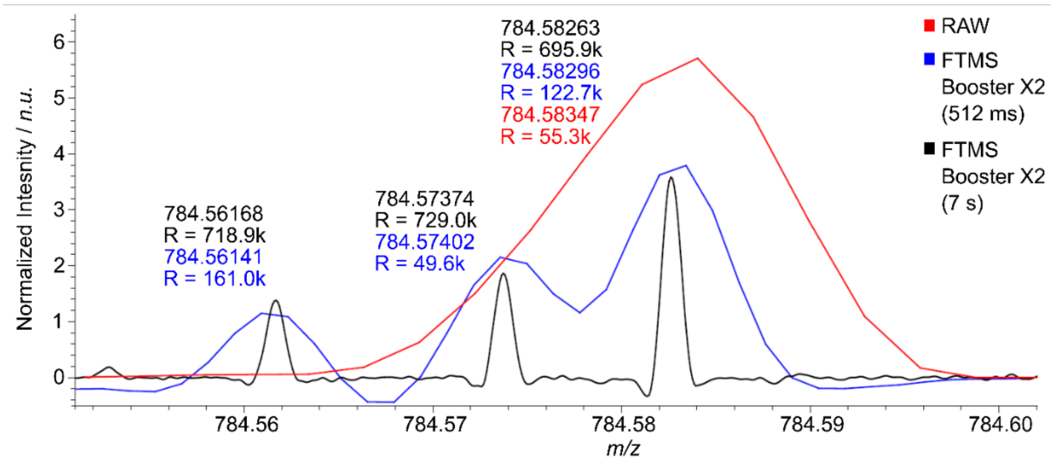

Figure S9. An overlay of MALDI mass spectra acquired in a positive ion mode using the FTMS Booster X2 (512 ms acquisition time (blue) and 7 s acquisition time (black)) and the stock QE HF digitizer to RAW data (red, 256 ms acquisition time).

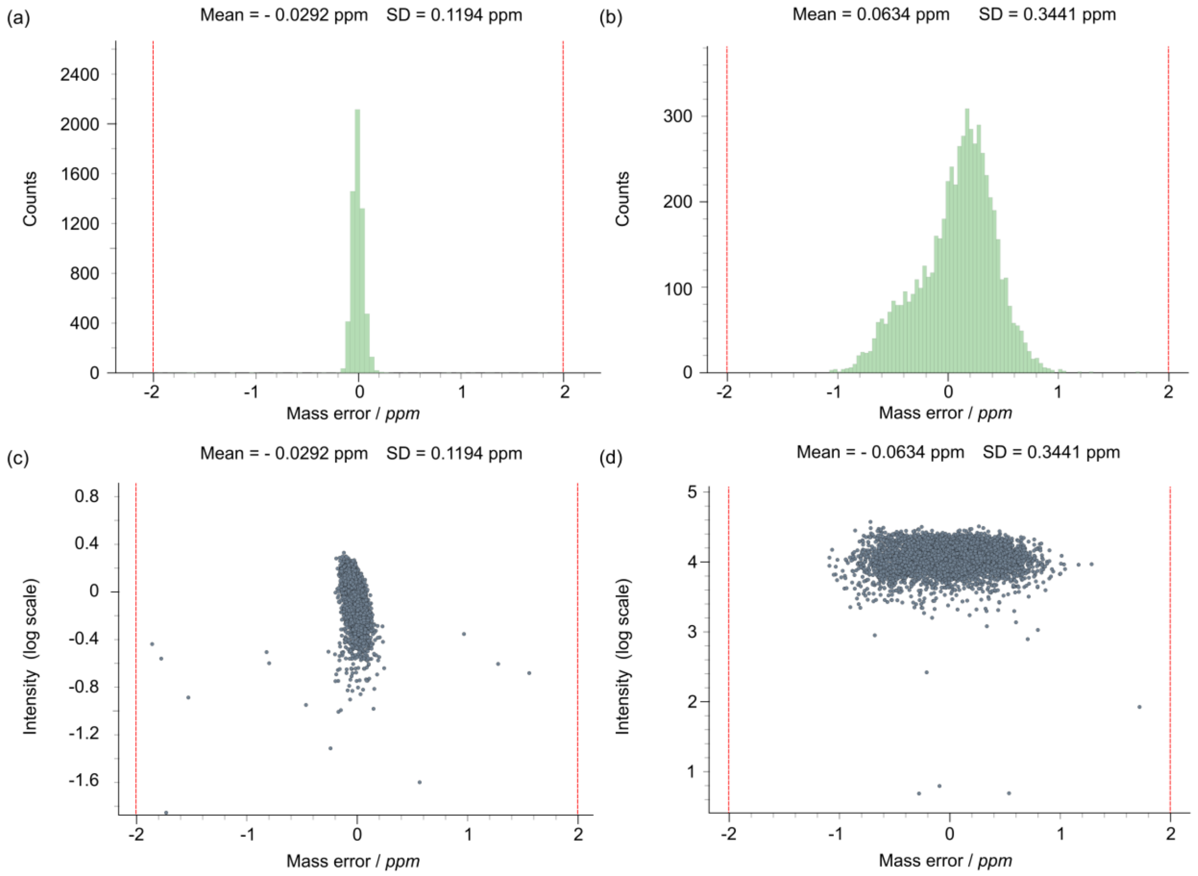

Figure S10. (**a**) Spread of mass errors observed for a peak at 798.54097 *m/z* in unreduced data with a transient length of 7 seconds. (**b**) Spread of mass errors observed for a peak at 798.54095 *m/z* in RAW data with a transient length of 256 ms. (**c**) Mass error vs. Intensity plot in unreduced data with transient length of 7 seconds (**d**) Mass error vs. Intensity plot in RAW data with transient length of 256 ms. Both RAW data and aFT data have been recalibrated using the reference list provided in Table S2a.

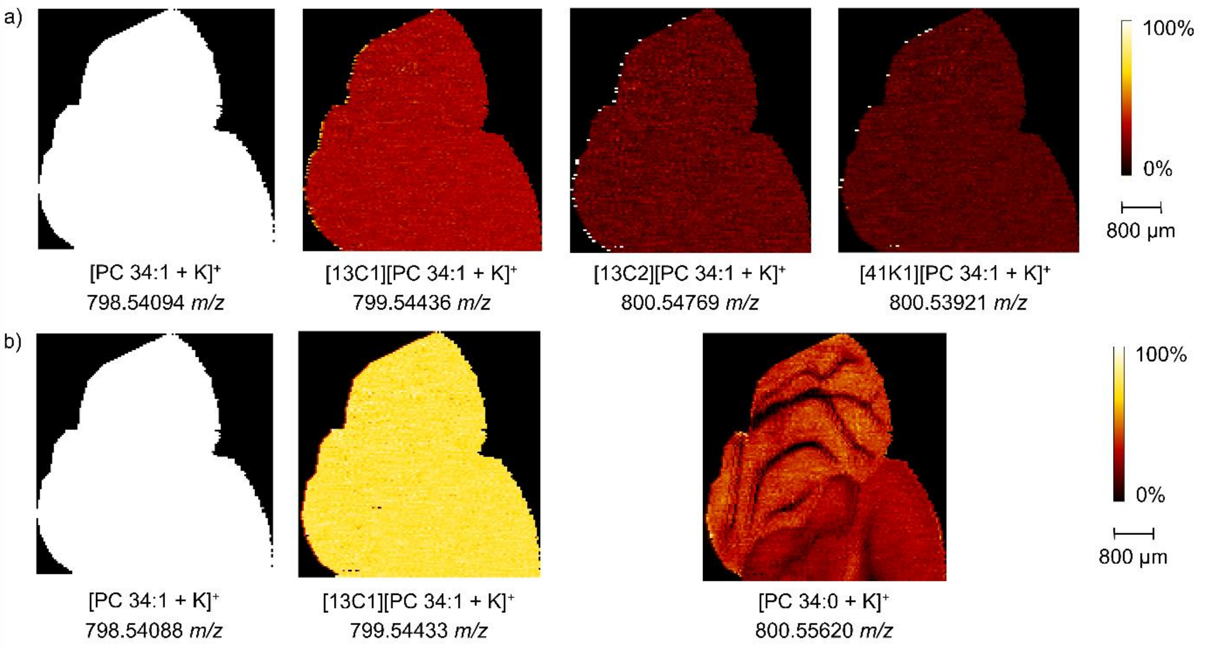

Figure S11. Images of $[PC\ 34:1 + K]^+$ and corresponding isotopic peaks normalized to the intensity of the monoisotopic ion at *m/z* 798.54094 for unreduced data and *m/*z 798.54088 for RAW data. (**a**) Images generated with the FTMS Booster X2 acquired data set. (**b**) Images generated with the RAW (eFT) data set collected by the in-built DAQ unit of the Orbitrap QE HF. As the peak at 800.5 *m/z* remains unresolved, the generated image represents the combined spatial distribution of $[41K][PC\ 34:1 + K]^+$, $[13C2][PC\ 34:1 + K]^+$, and $[PC\ 34:0 + K]^+$.

Images of $[PC\ 34:1 + K]^+$ are white due to the normalization of mass spectra at each pixel relative to the intensity of $[PC\ 34:1 + K]^+$. Consequently, the intensity of this lipid in each pixel corresponds to 1 (or 100%). As mass spectrum interferences remain unresolved in the RAW data, the intensity of the M+1 peak is artificially higher than the intensity of the same peak in the unreduced data. Finally, while M+2 peaks are fully resolved in the unreduced data, images generated from the RAW data correspond to spatial distributions of $[41K1][PC\ 34:1 + K]^+$, $[13C2][PC\ 34:1 + K]^+$, and $[PC\ 34:0 + K]^+$, reflecting their unresolved nature due to insufficient mass resolution.

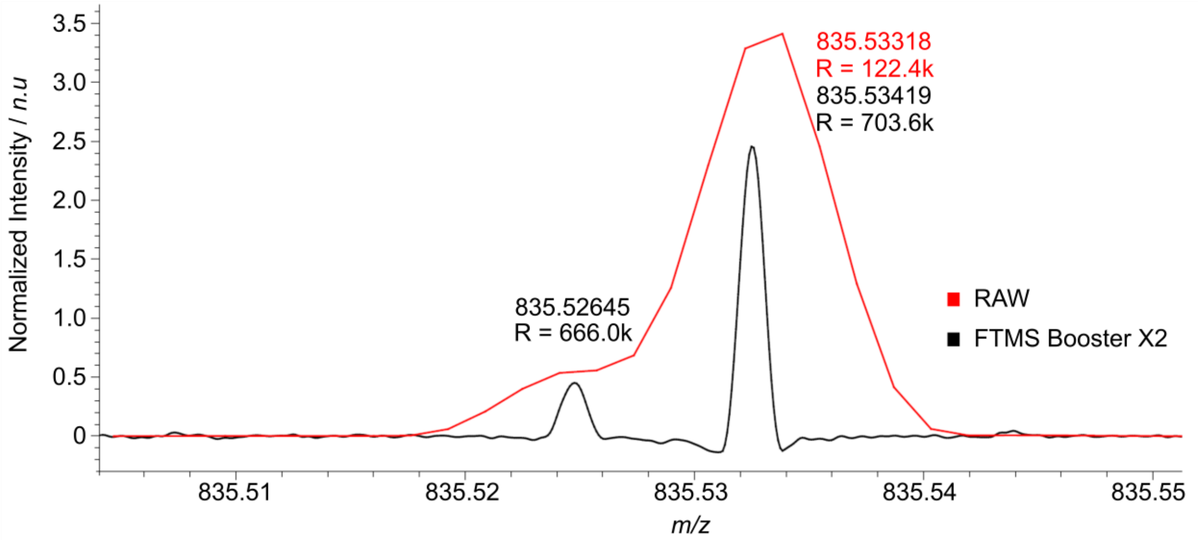

Figure S12. An overlay of MALDI mass spectra acquired in a negative ion mode using the FTMS Booster X2 (black, 7 s acquisition time) and the stock QE HF digitizer to RAW data (red, 512 ms acquisition time). As shown in Figure 4, resolution of the isobaric peak at *m/z* 835.53318 results in two distinct ion signals with different spatial distributions.

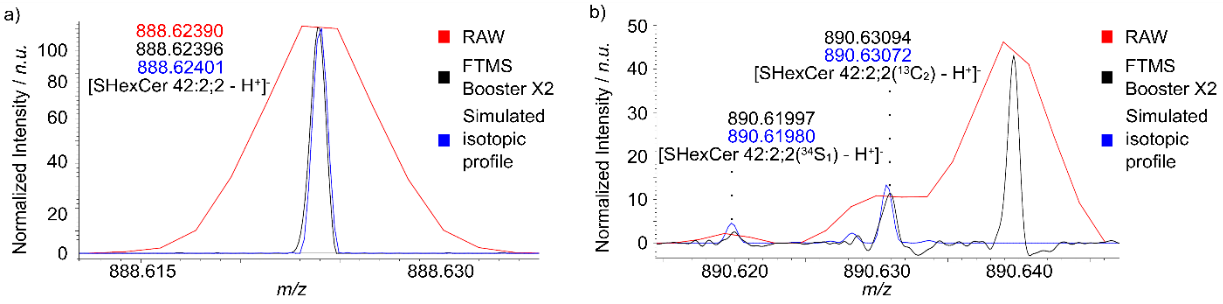

Figure S13. Mass spectrum of SHexCer 42:2;2 and corresponding M+2 isotopic peaks. (**a**) The molecular peak of [SHexCer 42:2;2 - H+]- (**b**) Overlay of acquired mass spectra with a simulated isotopic profile in FTMS Simulator (Mozaic software).[7]

References

(1) Nagornov, K. O.; Tsybin, O. Y.; Nicol, E.; Kozhinov, A. N.; Tsybin, Y. O. Fourier Transform Ion Cyclotron Resonance Mass Spectrometry at the True Cyclotron Frequency. *Mass Spectrom. Rev.* **2022**, *41* (2), 314–337. https://doi.org/10.1002/mas.21681.

(2) Xie, Y. R.; Castro, D. C.; Rubakhin, S. S.; Sweedler, J. V; Lam, F. Enhancing the Throughput of FT Mass Spectrometry Imaging Using Joint Compressed Sensing and Subspace Modeling. *Anal. Chem.* **2022**, *94* (13), 5335–5343. https://doi.org/10.1021/acs.analchem.1c05279.

(3) Ellis, S. R.; Paine, M. R. L.; Eijkel, G. B.; Pauling, J. K.; Husen, P.; Jervelund, M. W.; Hermansson, M.; Ejsing, C. S.; Heeren, R. M. A. Automated, Parallel Mass Spectrometry Imaging and Structural Identification of Lipids. *Nat. Methods* **2018**, *15* (7), 515–518. https://doi.org/10.1038/s41592-018-0010-6.

(4) Scigelova, M.; Hornshaw, M.; Giannakopulos, A.; Makarov, A. Fourier Transform Mass Spectrometry. *Mol. Cell. Proteomics* **2011**, *10* (7), 1–19. https://doi.org/10.1074/mcp.M111.009431.

(5) Qi, Y.; Li, H.; Wills, R. H.; Perez-Hurtado, P.; Yu, X.; Kilgour, D. P. A.; Barrow, M. P.; Lin, C.; O'Connor, P. B. Absorption-Mode Fourier Transform Mass Spectrometry: The Effects of Apodization and Phasing on Modified Protein Spectra. *J. Am. Soc. Mass Spectrom.* **2013**, *24* (6), 828–834. https://doi.org/10.1007/s13361-013-0600-6.

(6) Zhurov, K. O.; Kozhinov, A. N.; Fornelli, L.; Tsybin, Y. O. Distinguishing Analyte from Noise Components in Mass Spectra of Complex Samples: Where to Cut the Noise? *Anal. Chem.* **2014**, *86* (7), 3308–3316. https://doi.org/10.1021/ac403278t.

(7) Nagornov, K. O.; Kozhinov, A. N.; Gasilova, N.; Menin, L.; Tsybin, Y. O. Transient-Mediated Simulations of FTMS Isotopic Distributions and Mass Spectra to Guide Experiment Design and Data Analysis. *J. Am. Soc. Mass Spectrom.* **2020**, *31* (9), 1927–1942. https://doi.org/10.1021/jasms.0c00190.
